# scAce: an adaptive embedding and clustering method for single-cell gene expression data

**DOI:** 10.1093/bioinformatics/btad546

**Published:** 2023-09-06

**Authors:** Xinwei He, Kun Qian, Ziqian Wang, Shirou Zeng, Hongwei Li, Wei Vivian Li

**Affiliations:** School of Mathematics and Physics, China University of Geosciences, Wuhan 430074, China; School of Mathematics and Physics, China University of Geosciences, Wuhan 430074, China; School of Mathematics and Physics, China University of Geosciences, Wuhan 430074, China; School of Mathematics and Physics, China University of Geosciences, Wuhan 430074, China; School of Mathematics and Physics, China University of Geosciences, Wuhan 430074, China; Department of Statistics, University of California, Riverside, Riverside 92521, United States

## Abstract

**Motivation:**

Since the development of single-cell RNA sequencing (scRNA-seq) technologies, clustering analysis of single-cell gene expression data has been an essential tool for distinguishing cell types and identifying novel cell types. Even though many methods have been available for scRNA-seq clustering analysis, the majority of them are constrained by the requirement on predetermined cluster numbers or the dependence on selected initial cluster assignment.

**Results:**

In this article, we propose an adaptive embedding and clustering method named scAce, which constructs a variational autoencoder to simultaneously learn cell embeddings and cluster assignments. In the scAce method, we develop an adaptive cluster merging approach which achieves improved clustering results without the need to estimate the number of clusters in advance. In addition, scAce provides an option to perform clustering enhancement, which can update and enhance cluster assignments based on previous clustering results from other methods. Based on computational analysis of both simulated and real datasets, we demonstrate that scAce outperforms state-of-the-art clustering methods for scRNA-seq data, and achieves better clustering accuracy and robustness.

**Availability and implementation:**

The scAce package is implemented in python 3.8 and is freely available from https://github.com/sldyns/scAce.

## 1 Introduction

Advances in single-cell RNA sequencing (scRNA-seq) technologies have made them powerful tools for understanding heterogeneous gene expression in diverse cell populations and for quantifying single-cell activities in the study of development, physiology, and disease. In computational analysis of scRNA-seq data, unsupervised clustering is a crucial approach for identifying distinct cell populations based on their gene expression levels (Kiselev *et al.* 2019, Sheng and Li 2021, Li 2022). By unsupervised clustering, it is possible to identify clusters of cells and then annotate them as known or novel cell types based on prior knowledge of marker genes and biological pathways. However, due to the high sparsity and high-dimensional nature of scRNA-seq data (Petegrosso *et al.* 2020, Qi *et al.* 2020), it is challenging to cluster single cells directly using generic clustering methods.

To better account for the characteristics of scRNA-seq data, new clustering methods tailored for single-cell gene expression levels have been developed. Earlier methods, such as Seurat (Satija *et al.* 2015), SC3 (Kiselev *et al.* 2017), and CIDR (Lin *et al.* 2017), treat dimensionality reduction and cell clustering as two successive steps. They first use principal component analysis to reduce the dimensions of the gene expression matrix or the cell-cell distance matrix, and then utilize a generic clustering method, such as the Louvain (Blondel *et al.* 2008) or hierarchical clustering (Ward *et al.* 1963) to obtain the inferred cluster labels. More recent methods such as scScope (Deng *et al.* 2019) use an autoencoder, a deep-learning-based model, to learn low-dimensional latent representation of data and then perform cell clustering on the low-dimensional features. Another example is graph-sc (Ciortan and Defrance 2022), which uses a convolutional graph autoencoder to process a gene-to-cell graph before applying the K-means or Leiden (Traag *et al.* 2019) clustering algorithm. Since the latent space learned by traditional autoencoders is discontinuous and unregularized, deep embedding methods based on variational autoencoder (VAE) networks have gained popularity in scRNA-seq analysis. VAEs can learn a continuous latent representation of the input data by constraining the distribution of the latent variables to follow a prior distribution. VAE-based methods such as VASC (Wang and Gu 2018) and siVAE (Choi *et al.* 2023) focus more on the cell embedding problem, while other methods such as scVI (Lopez *et al.* 2018), scVAE (Grønbech *et al.* 2020), and scGMAAE (Wang *et al.* 2023) can simultaneously achieve cell embedding and clustering.

As most clustering methods for scRNA-seq data rely on external or internal dimensionality reduction as an intermediate step, the quality of the low-dimensional representation has significant impact on the downstream clustering accuracy. Instead of treating dimensionality reduction and clustering as two separate tasks, some clustering methods based on deep embedding aim to simultaneously learn low-dimensional embeddings and clusters (Xie *et al.* 2016, Guo *et al.* 2017). By adapting this idea of deep embedding clustering to cluster single cells, multiple methods have been proposed for scRNA-seq data. For example, DESC (Li *et al.* 2020) applies deep embedding clustering to scRNA-seq data after normalization and gene selection, with cluster centers initialized by the Louvain algorithm. scDeepCluster (Tian *et al.* 2019) and scDCC (Tian *et al.* 2021) extended this idea by incorporating a zero-inflated negative binomial (ZINB) model, which was first proposed in the DCA method for scRNA-seq denoising (Eraslan *et al.* 2019), into an autoencoder network. Even though these methods based on deep embedding sometimes lead to better clustering results by allowing for nonlinear transformations, one limitation they share is that their optimization procedure depends on cell clusters initialized by a generic algorithm such as K-means or Louvain. The K-means algorithm requires a cluster number as input to perform clustering, while the Louvain algorithm requires a resolution parameter to control the size of the clusters. If these parameters are mis-specified or if the initial clustering results contain too many incorrect mixtures of cell populations, these errors will propagate to the iterative update of neural networks, affecting the accuracy of final clustering results.

In light of the above limitation, some clustering methods attempt to reduce the dependence on predetermined parameters (such as cluster number) by starting with a relatively large number of micro clusters and gradually merging similar ones into larger clusters. For example, both SCCAF (Miao *et al.* 2020) and ADClust (Zeng *et al.* 2022) obtain initial clusters via the Louvain algorithm. Then, SCCAF iteratively updates the cluster labels by training a classifier on the clusters and evaluating the similarities between the clusters based on a confusion matrix; ADClust uses a unimodality test to evaluate the similarity between clusters and identify those that could be merged. Although cluster merging has reduced their reliance on the initial cluster number, as we will show in our results, their merging process uses non-data-adaptive stopping criteria and could be prone to under-clustering or over-clustering on certain datasets.

Inspired by these preceding endeavors in clustering analysis, we propose a method named scAce for scRNA-seq data to simultaneously achieve embedding of gene expression data and clustering of single cells. The scAce method constructs a VAE network to learn smoother low-dimensional embeddings compared with those methods based on traditional autoencoders. It utilizes a data-adaptive clustering approach based on the idea of cluster merging, and the merging process is controlled by evaluating intra-cluster and inter-cluster distances. By iteratively updating the VAE network and the cluster labels, scAce improves both the embedding and clustering of single cells. Another feature of the scAce method is that it enables clustering enhancement by taking the clustering results of another method as its initialization, and then uses its network model to further enhance the accuracy of final clusters. We have assessed the clustering performance of scAce in comparison with state-of-the-art clustering methods. The results show that scAce is more accurate and robust on both simulated and real scRNA-seq data. In addition, with its cluster enhancement option, scAce is able to correct and improve previous clustering results produced by other clustering methods.

## 2 Materials and methods

### 2.1 An outline of the scAce method

**Figure 1.**
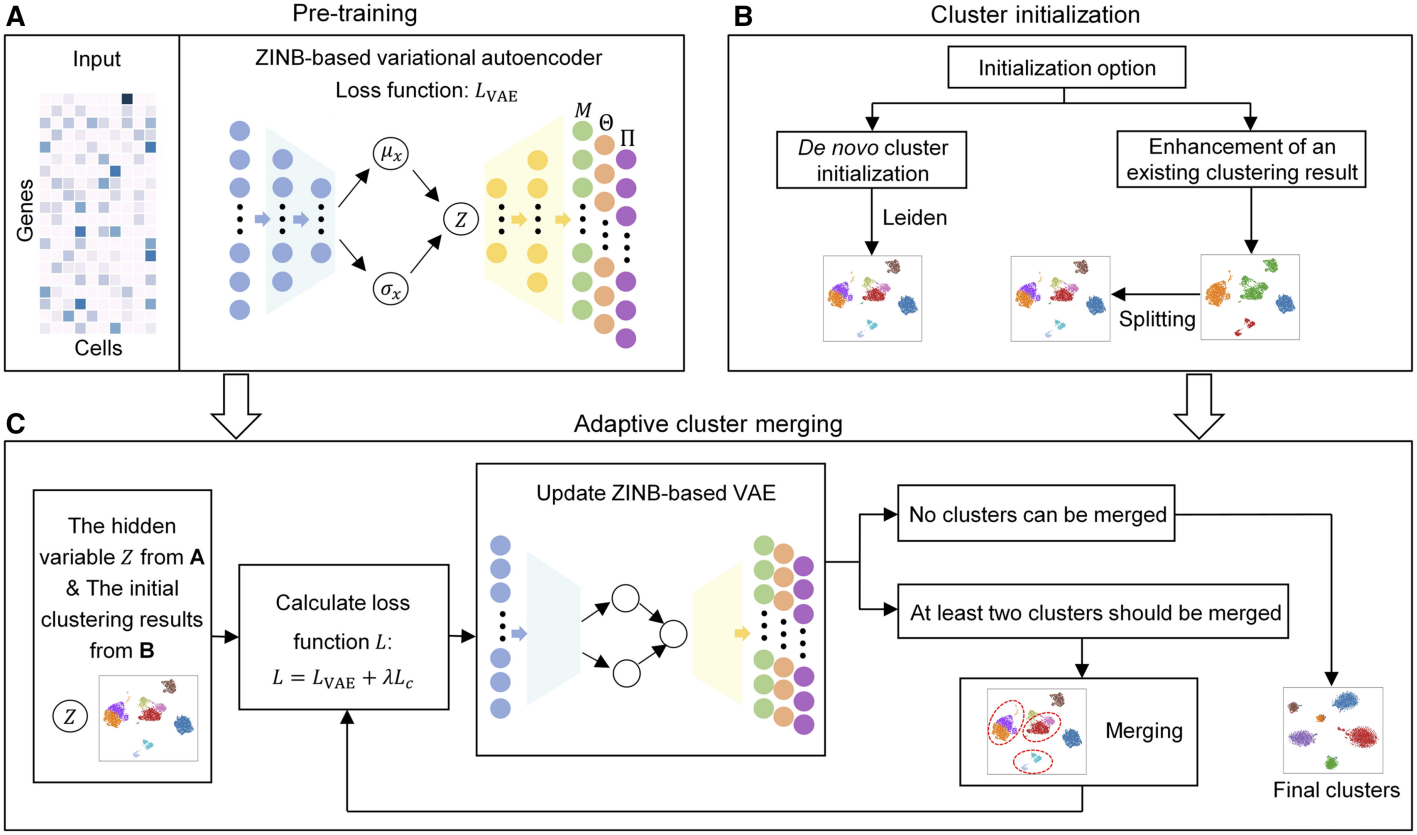
Overview of the scAce method. (A) Pretraining. scAce takes the single-cell gene expression matrix as its input to train a VAE network. The encoder learns low-dimensional hidden variables for the single cells, which serve as the input of the decoder. For each gene, the VAE learns and outputs three parameters of a ZINB distribution (mean, dispersion, and inflated proportion of zero). (B) Cluster initialization. With *de novo* initialization, the Leiden algorithm is used to obtain initial cluster labels. With clustering enhancement, initial cluster labels are obtained by applying a cluster splitting approach to a set of existing clustering results (from another clustering method). (C) Adaptive cluster merging. Given the pretrained VAE network and the initial cluster labels, the network parameters, cell embeddings, cluster labels, and centroids are iteratively updated by alternately performing network update and cluster merging steps. The final results of cell embeddings and cluster labels are output by scAce after the iteration process stops.

The scAce method is consisted of three major steps, a pretraining step based on an improved variational autoencoder (*β*-VAE) (Higgins *et al.* 2017), a cluster initialization step to obtain initial cluster labels, and an adaptive cluster merging step to iteratively update cluster labels and cell embeddings (Fig. 1). We introduce each of these steps in detail below.

***Pretraining***. The input of scAce is a scRNA-seq read count matrix $X\in R^{m\times n}$, based on which scAce will obtain the normalized expression matrix $\tilde{X}\in R^{m\times n}$ (Supplementary Methods), where *m* and *n* are the numbers of genes and cells after preprocessing, respectively. In the VAE, the expression levels are first encoded to obtain the mean and variance parameters in the hidden layer. Then, the decoder receives a hidden variable $Z\in R^{d\times n}(d\ll m)$ generated in the lower-dimensional space produced by the encoder. Based on *Z*, the decoder learns three parameters (mean, dispersion, and inflated zero proportion) of a ZINB distribution for every gene (Fig. 1A).

***Cluster initialization***. scAce provides two options to perform cluster initialization (Fig. 1B). The first option is used when scAce is applied in a *de novo* manner, and an existing clustering result is not available. In this case, scAce uses the Leiden algorithm in Scanpy (Wolf *et al.* 2018) to obtain the initial cluster assignments and centroids. For each cluster, the initial cluster centroid is the center of the cluster based on the hidden variable *Z* obtained from the pretraining step and the Euclidean distance. The resolution parameter of the Leiden algorithm is set to a large value (defaults to 2) so that the clusters have high purity. The second option is used when enhancement of an existing clustering result (e.g. obtained using Seurat) is desired. When using this option, scAce takes the cluster labels from the existing clustering result, and applies an adaptive cluster splitting method (see Supplementary Methods) to split the current clusters into smaller and purer clusters based on intra-cluster distances. Then, scAce uses the new clusters after cluster splitting as the initial cluster assignment and obtains cluster centroids as described above.

The purpose of cluster splitting in clustering enhancement or using a large resolution parameter in the *de novo* initialization is to ensure that each of the initial clusters contains cells of the same cell type or state. Even though there might be multiple clusters in the initial assignment that belong to the same cell types, they will be merged into the same cluster at the adaptive cluster merging step. This approach was inspired by the observation that methods depending on initial clustering results of the K-means or Louvain algorithm are likely to generate mixed clusters if the initial ones contain a large proportion of mixtures.

***Adaptive cluster merging***. In the step of adaptive cluster merging, scAce iteratively performs network update and cluster merging based on the initial VAE network obtained from the pretraining step and the clusters from the cluster initialization step (Fig. 1C). At each iteration of network update, scAce constructs a loss function consisted of two components. The first component is the loss of the VAE network and the second component is a clustering loss defined based on cluster labels and centroids. Given this loss function, the VAE network is updated to improve cell embeddings. At each iteration of cluster merging, scAce decides if a pair of clusters should be merged into a single cluster by comparing inter-cluster and intra-cluster distances. Network update and cluster merging are performed alternately in scAce until no clusters can be merged in the cluster merging step.

### 2.2 Pretraining: ZINB-based variational autoencoder network

Since the latent space learned by traditional autoencoders are discontinuous and unregularized, which is not ideal for generative modeling, we use a VAE network with a Gaussian prior (Kingma *et al.* 2022) to learn the latent space of single-cell gene expression data. In addition, we use ZINB as the generative distribution in the decoder to model the scRNA-seq count data. For gene *i* in cell *j* (i=1,…,m,j=1,…,n), we assume that its count follows a ZINB distribution with the following parameterization:

(1) $$\text{ZINB}(x;\mu_{ij},\theta_{ij},\pi_{ij})=\pi_{ij}\delta_{0}(x)+(1-\pi_{ij})\text{NB}(x;\mu_{ij},\theta_{ij}),$$

where $\text{NB}(x;\mu_{ij},\theta_{ij})$ denotes a negative binomial (NB) distribution; *μ_ij_* and *θ_ij_* are the mean and dispersion parameters of the NB distribution, respectively; $\delta_{0}(x)$ is the Dirac delta function which takes the values of 1 when *x *=* *0 and 0 when x≠0; *π_ij_* denotes the inflated zero proportion.

We now introduce the architecture of the VAE network in detail (Fig. 1A). Given the normalized single-cell gene expression matrix $\tilde{X}\in R^{m\times n}$, the encoder first obtains the mean and variance parameters of the Gaussian distributions in the hidden space. Then, the network uses the resampling technique to obtain the hidden Gaussian variables $Z\in R^{d\times n}$, which serves as the input of the decoder. Lastly, the output of the decoder are the parameters of the ZINB distributions. We denote the mean parameters as $M≜[\mu_{ij}]\in R^{m\times n}$, the dispersion parameters as $\Theta ≜[\theta_{ij}]\in R^{m\times n}$, and the inflated zero proportions as $\Pi ≜[\pi_{ij}]\in R^{m\times n}$. The VAE model is therefore summarized as follows:

(2) $$\{\begin{array}{cc} & H=f_{H}(\tilde{X});\;\;\;\;\mu_{x}=W_{\mu_{x}}H;\\ & \;\text{log}\;\sigma_{x}=W_{\sigma_{x}}H;\;\;Z=\sigma_{x}⊙\epsilon +\mu_{x};\\ & D=f_{D}(Z);\;\;\;\;M=S⊙\;\text{exp}(W_{\mu }D);\\ & \Theta =\text{exp}(W_{\theta }D);\;\;\Pi =\text{sigmoid}(W_{\pi }D) \end{array}\;,$$

where:

-
$\tilde{X}\in R^{m\times n}$ is the input normalized gene expression matrix;
-
$H\in R^{d_{1}\times n}(d<d_{1}<m)$ is the output of $f_{H}(\cdot )$, where $f_{H}(\cdot )$ is a layer of fully connected neural network with the ReLU activation function;
-
$\mu_{x}\in R^{d\times n}$ and $\sigma_{x}\in R^{d\times n}$ are the mean and standard deviation parameters of the hidden Gaussian variables; $W_{\mu_{x}}\in {\mathbb{R}}^{d\times d_{1}}\;\text{and}\;W_{\sigma_{x}}\in {\mathbb{R}}^{d\times d_{1}}$ are the corresponding weights;
-
$Z\in R^{d\times n}$ is drawn from the learned Gaussian distributions and serves as the input of the decoder $f_{D}(\cdot )$; each element in $\epsilon \in R^{d\times n}$ is an independent standard Gaussian variable, and ⊙ denotes the element-wise multiplication;
-
$f_{D}(\cdot )$ is a layer of fully connected neural network with the ReLU activation function;
-
$D\in R^{d_{2}\times n}(d<d_{2}<m)$ is the output of the decoder $f_{D}(\cdot )$; $W_{\mu }\in R^{m\times d_{2}},W_{\theta }\in R^{m\times d_{2}},\;\text{and}\;W_{\pi }\in R^{m\times d_{2}}$ are the weight parameters.

In this model, we use the exponential activation function to generate the mean parameters (*M*) and dispersion parameters (Θ) because these parameters should be nonnegative. We also use the sigmoid activation function to calculate Π so that the values are between 0 and 1. Given this model, the loss function of the VAE network is derived as

(3) $$\begin{array}{cc} L_{\text{VAE}}= & -\frac{1}{n}\sum_{i=1}^{m}\sum_{j=1}^{n}\;\text{log}\;\;\text{ZINB}(x_{ij},\mu_{ij},\theta_{ij},\pi_{ij})\\ & -\frac{\beta }{2n}\sum_{i=1}^{d}\sum_{j=1}^{n}(1+\text{log}(\sigma_{x}{)}_{ij}^{2}-{\left(\mu_{x}\right)}_{ij}^{2}-{\left(\sigma_{x}\right)}_{ij}^{2}). \end{array}$$

The first term in $L_{\text{VAE}}$ represents the negative log-likelihood function, and the second term is derived from the Kullback–Leibler divergence KL(q(Z|X)||p(Z)) (Supplementary Methods). β≥1 is an adjustable parameter that reflects the strength of the disentanglement constraint.

### 2.3 Adaptive cluster merging

Given results of the pretraining and cluster initialization steps, scAce first performs a network update, and then iteratively performs cluster merging and network update to obtain the final clustering results (Fig. 1C). The network update step and the cluster merging step are performed alternately, and the iteration stops if no clusters can be merged after a network update step. The final cluster labels and cell embeddings are the output of scAce.

At each step of network update, given the current cluster labels, scAce updates the VAE network parameters and cluster centroids using the deep embedded clustering technique. A loss function is constructed to represent the quality of the clustering results based on the current cell embeddings and cluster labels. This function is then combined with the loss function of the VAE network to simultaneously update the network parameters and cluster centroids. After the update, scAce switches to the cluster merging step.

At each step of cluster merging, given the current cell embeddings (from updated VAE network) and cluster centroids, scAce first assigns cells to their closest centroids to update the cluster labels. Then, using a data-adaptive criterion, scAce merges pairs of clusters with highly similar gene expression profiles into the same cluster. When two clusters are merged, the new centroid of the merged cluster is updated as the center of that cluster based on the current data embedding. The merging process is repeated until no more clusters can be merged given the data-adaptive criterion. Then, scAce switches to the network update step.

***Network update***. Suppose at the beginning of a network update step there are *K* clusters and corresponding centroids. We calculate the probability that cell i (i=1,2,…,n) belongs to cluster j (j=1,2,…,K) as the conditional probability ($q_{i|j}$) of cell *i* given the centroid of cluster *j* (denoted as *c_j_*). The conditional probability is obtained using the student’s *t*-distribution as a kernel to measure the similarity between the embedded cells and cluster centroids (Xie *et al.* 2016):

(4) $$q_{i|j}=\frac{{\left(1+||z_{i}-c_{j}|{|}^{2}\right)}^{-1}}{\sum_{j\prime =1}^{K}{\left(1+||z_{i}-c_{j\prime }|{|}^{2}\right)}^{-1}}\;.$$

To improve cluster quality by putting more emphasis on cells assigned with a high confidence, we define an auxiliary target distribution based on the distribution represented by the conditional probabilities:

(5) $$p_{i|j}=\frac{q_{i|j}^{2}/f_{j}}{\sum_{j\prime =1}^{K}q_{i|j\prime }^{2}/f_{j\prime }},$$

where $f_{j}=\sum_{i=1}^{n}q_{i|j}$. The model is then trained to increase the similarity between the current distribution and the target distribution. The goal is to minimize the sum of the KL divergence over all the cells:

(6) $$L_{C}=\sum_{i=1}^{n}\sum_{j=1}^{K}p_{i|j}\;\text{log}\;\frac{p_{i|j}}{q_{i|j}}.$$

Then, in order to update the network parameters, the overall loss function is defined as a weighted sum of $L_{\text{VAE}}$ and *L_C_*: $L=L_{\text{VAE}}+\lambda L_{C},$ where λ>0 is a parameter controlling the relative weights of network loss and clustering loss. By minimizing the loss function *L*, the network parameters are optimized, and the embeddings of the single cells and cluster centroids are updated, thus updating the cluster labels. After the cluster labels are updated, we recalculate the conditional probabilities in Equation (4) and repeat the above process to update cluster labels until the proportion of cells that change their cluster labels between two consecutive repeats is smaller than 5%. The purpose of this repeating process is to ensure that the network update has reached a stable state before cluster merging.

***Cluster merging***. Given the updated data from the previous network update step, we use a data-adaptive criterion to decide if two smaller clusters should be merged into a larger cluster (Lei *et al.* 2016). To define this criterion, we first introduce the intra-cluster distance. Suppose there are *K* clusters in a given iteration. For the *i*th cluster (i=1,…,K), its intra-cluster distance is defined as

(7) $$d_{i}^{\text{intra}}=\frac{1}{\left|N_{i}\right|}\sum_{q\in N_{i}}||z_{q}-c_{i}||,$$

where *N_i_* denotes the indices of cells in cluster *i*; $\left|N_{i}\right|$ is the number of cells in cluster *i*; *c_i_* denotes the centroid of cluster *i*; *z_q_* is the current embedding of cell *q*. In addition, we define the inter-cluster distance between clusters *i* and *j* as

(8) $$d_{ij}^{\text{inter}}=||c_{i}-c_{j}||,$$

where *c_i_* and *c_j_* denote the centroids of clusters *i* and *j*, respectively.

The rationale underlying the adaptive criterion is that two small clusters should be merged into a larger cluster when their inter-cluster distance is small compared with the average inter-cluster distance defined as

(9) $$\bar{d}=\frac{2}{K(K-1)}\sum_{i=1}^{K}\sum_{j=i+1}^{K}w_{ij}d_{ij}^{\text{inter}},$$

where *w_ij_* is the assigned weight on the inter-cluster distance between the *i*th and the *j*th clusters. This weight is used to account for the effect of cluster size on inter-cluster distances, and is defined as

(10) $$w_{ij}=\frac{{\bar{d}}^{\text{intra}}}{\frac{1}{2}\left(d_{i}^{\text{intra}}+d_{j}^{\text{intra}}\right)}=\frac{\frac{1}{K}\sum_{i=1}^{K}d_{i}^{\text{intra}}}{\frac{1}{2}\left(d_{i}^{\text{intra}}+d_{j}^{\text{intra}}\right)}.$$

For example, if the size of cluster *i* is large, then it will naturally have relatively large inter-cluster distances with other clusters. Consequently, it will have relatively smaller weights to offset this effect. In summary, we find all cluster pairs whose weighted intra-cluster distance ($w_{ij}d_{ij}^{\text{inter}})$ is smaller than $\bar{d}/2$ and merge the cluster pair with the smallest weighted distance.

Following this principle, after each merge, Equations (7–10) are updated based on the new cluster memberships, and the merging process is repeated until no more clusters meet the merging criterion. In other words, we stop the cluster merging step when $w_{ij}d_{ij}^{\text{inter}}>\bar{d}/2$ for any pairs of *i* and *j* (i≠j).

### 2.4 Implementation

The scAce package is implemented in python 3.8, using Scanpy version 1.9.1 (Wolf *et al.* 2018) for preprocessing and Pytorch version 1.10.0 for implementing the VAE network. In the ZINB-based VAE network, *β* is set to 0.001×m during pretraining and 0.01×m during adaptive cluster merging. The values of $d_{1},d,d_{2}$ were set to 512, 32, and 512 in our analysis, and these are provided as tuning parameters in the scAce software. The parameter *λ* is set to 1 by default. A comparison for different values of *λ* and *β* at the adaptive cluster merging stage is presented in Supplementary Tables S1 and S2. The required input of the scAce software is a scRNA-seq read count matrix. If clustering enhancement of an existing clustering result is desired, then the optional input of existing cluster labels should also be provided. The output of scAce includes the low-dimensional embeddings and final cluster assignments of the single cells.

### 2.5 Clustering methods used for comparison

For comparison in performance evaluation, we considered nine alternative clustering methods developed for scRNA-seq data based on both citation number and publication time. We restricted our considerations to methods that have software functions to directly perform clustering. From the traditional clustering methods (not using deep-learning methods), we selected Seurat (Satija *et al.* 2015) and CIDR (Lin *et al.* 2017), both of which are highly cited. From the clustering methods based on classical autoencoders, we selected the most widely used scDeepCluster (Tian *et al.* 2019) and DESC (Li *et al.* 2020). From clustering methods based on VAEs, we chose the most widely used scVI method (Lopez *et al.* 2018) and the recently published scGMAAE method (Wang *et al.* 2023). In addition, we selected two clustering methods that also use cluster merging approaches, SCCAF (Miao *et al.* 2020) and ADClust (Zeng *et al.* 2022), and one clustering method based on the graph neural network, graph-sc (Ciortan and Defrance 2022). For methods requiring a cluster number as input, the number of real cell types was provided to the algorithms. The main characteristics of scAce and the other nine methods are summarized in Supplementary Table S3.

### 2.6 Datasets

For simulated data, we used the R package scDesign2 (Sun *et al.* 2021) to generate a synthetic single-cell gene expression matrix with ground truth cell type labels. In this simulated dataset, there were 16 653 genes and 1150 cells belonging to five cell types. The number of cells in each cell type was 600, 200, 200, 100, and 50, respectively. The last cell type accounted for <5% of the cells and was used to represent a rare cell type. The real scRNA-seq dataset used by scDesign2 to learn gene expression parameters was a peripheral blood mononuclear cell (PBMC) dataset generated by the 10x Genomics technology (Zheng *et al.* 2017).

For real data, we downloaded seven real scRNA-seq datasets with annotated cell type labels (Supplementary Table S4). The seven datasets included three human datasets, three mouse datasets, and one turtle dataset. For simplicity, the datasets are referred to as Human pancreas (Baron *et al.* 2016) (3605 cells), Human PBMC (Zheng *et al.* 2017) (4271 cells), Human kidney (Young *et al.* 2018) (5685 cells), Mouse ES (Klein *et al.* 2015) (embryonic stem, 2717 cells), Mouse hypothalamus (Chen *et al.* 2017) (12 089 cells), Mouse kidney (Adam *et al.* 2017) (3660 cells), and Turtle brain (Tosches *et al.* 2018) (18 664 cells).

## 3 Results

### 3.1 scAce improves clustering accuracy and robustness on both simulated and real data

To evaluate the performance of scAce in clustering scRNA-seq data, we first applied it and the other nine clustering methods on the simulated data. We generated data of five cell types by the scDesign2 package (Sun *et al.* 2021), which can learn gene expression parameters and gene–gene correlations from real data (Section 2). For methods that require a cluster number as input (scDeepCluster, scGMAAE, and graph-sc), we input the real cell type number; for other methods including scAce, the cluster numbers were automatically determined by the methods.

**Figure 2.**
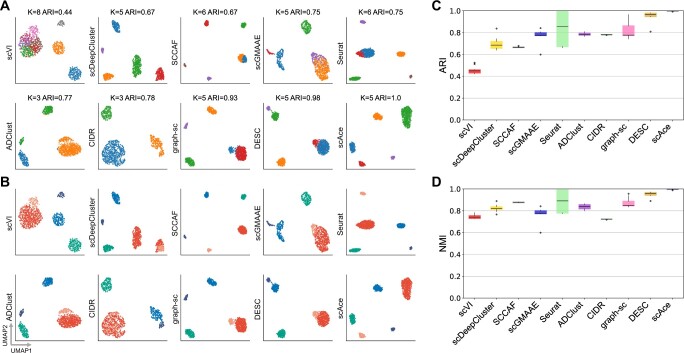
Comparison of the 10 clustering methods on the simulated dataset. (A) UMAP plots of the cell embeddings produced by the 10 methods. Each point represents a cell and each color represents an inferred cluster. The number of inferred clusters (*K*) and the ARI values of the clustering results are marked on top of the corresponding plots. (B) Same UMAP plots as shown in (A) but colored by the five true cell types. (C) Boxplots of ARI values obtained by applying the ten clustering methods to randomly selected subsamples of the complete simulated data. (D) Boxplots of NMI values obtained by applying the 10 clustering methods to randomly selected subsamples of the complete simulated data.

When comparing the clustering results (Fig. 2A) with the ground truth cell type labels (Fig. 2B), we found that scAce was the only method that achieved an ARI of 1 with a clear separation of the five cell types in the low-dimensional space. In contrast, scVI, SCCAF, and Seurat would divide some cell types into smaller clusters, while ADClust and CIDR were unable to distinguish the rare cell type from other major cell types. DESC and graph-sc also achieved a high ARI, but both methods misclassified a small proportion of cells from the rare cell type. It is also worth noting that among the seven methods which do not require a cluster number as input, scAce and DESC were the only two methods that identified the correct number of clusters.

In order to further evaluate the robustness of different methods, we repeatedly applied the methods to a subset of the simulated cells. Each time, we randomly chose 95% of the cells in the dataset to create a new dataset, and performed the clustering analysis on the new data. The experiments were repeated 10 times. scAce achieved the highest median ARI and NMI values among the ten methods (Fig. 2C and D), demonstrating its high robustness compared with alternative methods.

**Figure 3.**
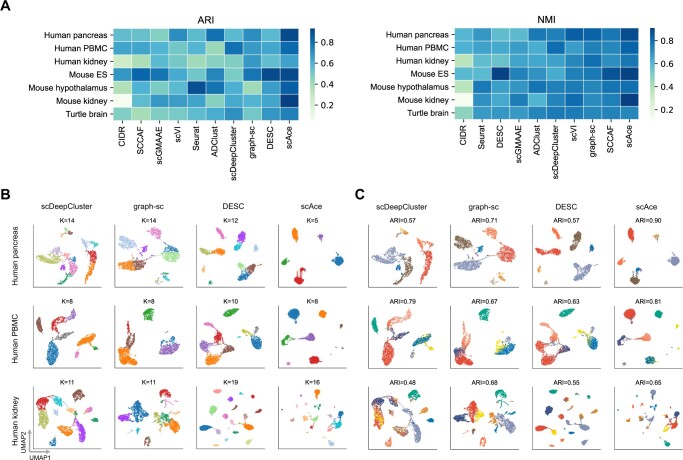
Comparison of clustering methods on real datasets. (A) ARI and NMI values obtained from the 10 methods on the seven real datasets. The methods are ordered based on the mean ARI/NMI values. (B) UMAP plots of the cell embeddings produced by the four methods with the highest average ARI values across datasets. Each point represents a cell and each color represents an inferred cluster. The inferred cluster numbers are marked on top of the corresponding plots. (C) Same UMAP plots as shown in (B) but colored by the true cell types. The ARI values of the clustering results are marked on top of the corresponding plots.

After confirming scAce’s accuracy on the simulated data, we then applied scAce and the other nine methods on seven real scRNA-seq datasets and evaluated their clustering accuracy (Section 2). Based on the mean ARI scores across the seven datasets, scAce achieved the best clustering accuracy, followed by DESC, graph-sc, scDeepCluster, and ADClust (Fig. 3A). The different cell types were clearly separated in the low-dimensional embeddings obtained by scAce, whereas the other methods had difficulty deriving embeddings that correctly reflected cellular identities for at least one dataset (Fig. 3B and C and Supplementary Figs S1 and S2). Compared with other clustering methods based on standard autoencoders (ADClust, DESC, and scDeepCluster), the embeddings obtained by scAce tended to be smoother, as VAE allows the latent variables to be continuous and smooth. When compared with scVI and scGMAAE, which also use VAEs to obtain cell embeddings, scAce’s clustering accuracy was higher than both on all seven datasets.

To further evaluate and compare the robustness of the clustering methods, we then applied them to subsets of 95% cells randomly selected from each real dataset, and repeated 10 times for each dataset. Based on both ARI and NMI scores, scAce again achieved the highest clustering accuracy across the repeated experiments (Supplementary Figs S3 and S4). For example, the average ARI of scAce across all experiments was 0.76, followed by graph-sc (average ARI = 0.66), and DESC (average ARI = 0.65). As for NMI, the average of scAce is 0.80, followed by DESC (average NMI = 0.78), and graph-sc (average NMI = 0.76).

### 3.2 scAce improves clustering accuracy by adaptive cluster merging

In order to investigate the necessity of the adaptive cluster merging step in scAce, we compared the clustering results of scAce (as described in Section 2) with the initial clustering results when setting the resolution parameter in the Leiden algorithm such that the initial cluster number was the same as the true cell type number. The ARI values under the two settings show that the results of scAce were more accurate than the initial clustering without performing adaptive cluster merging on six datasets (Supplementary Fig. S5A). The clustering accuracy of the two settings was similar on the Mouse ES dataset as the initial result already achieved an ARI close to 1 (Supplementary Fig. S5B). For the other more challenging datasets, scAce was able to achieve higher clustering accuracy even without prior knowledge about the true cell type number.

Of the nine alternative methods that were compared, SCCAF and ADClust also discover and merge clusters that might represent the same cellular identity in an iterative manner. In order to better compare the effectiveness and accuracy of the three methods, we studied the intermediate results of these methods in the initial and subsequent iterations. Their clustering results in the first iteration show that all three methods initially obtained a relatively large number of clusters (Supplementary Fig. S6A and B). Even though multiple clusters might correspond to the same cell type, most of these clusters had high purity, allowing for improvement by cluster merging through additional iterations.

We then compared how the correspondence between true cell types and identified clusters changed in the cluster merging process, using the Mouse kidney dataset as an example (Supplementary Figs S6 and S7). While the initial clustering of the three methods all showed high purity, the final clustering accuracy varied considerably. ADClust initially obtained 42 clusters, which were updated to 19 and 11 clusters in the second and third iteration, respectively. Even though each of these clusters only corresponded to a single cell type, starting from the fourth iteration, ADClust merged several groups of cells from different cell types into the same cluster. Its final result led to five clusters, all of which were mixtures of cells from multiple cell types. As for the SCCAF method, it stopped its merging process right after the initial iteration, leading to 17 clusters. It divided each of the eight cell types into multiple clusters, which made downstream annotations and comparisons error-prone. In contrast, scAce initially obtained 25 clusters, and gradually updated them to 17, 10, 9, and 8 clusters in the second to fifth iteration, respectively. Throughout this process, scAce was able to maintain the high purity of the identified clusters, and thus ultimately obtained clustering results that were in close agreement with the ground truth cell types (ARI = 0.93). Similar results were observed on the other six datasets (Supplementary Fig. S8).

To further investigate the observed advantages of scAce, we evaluated the performance of other clustering methods when they were combined with the same cluster initialization and cluster merging approaches as proposed in scAce. Among the nine alternative methods, we studied the four methods which can take initialized clusters as input (scDeepCluster, DESC, SCCAF, and ADClust), and performed cluster initialization as in scAce. After obtaining the clustering results from these methods, we then performed an additional cluster merging step as in scAce. Compared with scAce (average ARI = 0.82), the second and third best method in this comparison was scDeepCluster (average ARI = 0.72) and ADClust (average ARI = 0.61), respectively (Supplementary Fig. S9A). Our results show that simply combining the proposed cluster initialization and cluster merging approaches with other existing methods does not optimize the clustering results (Supplementary Figs S9 and S10). In contrast, the adaptive cluster merging approach used by scAce leads to improved results by combining cluster merging with network update in an iterative manner.

### 3.3 scAce is robust to cluster initialization

In the cluster initialization step, scAce uses the Leiden algorithm to obtain the initial clustering results. To evaluate the robustness of scAce to the selection of the initial clustering algorithm, we also applied scAce by using the Louvain or K-means algorithm to perform cluster initialization. The resolution parameter in Louvain was set to 2. For the cluster number in K-means, we tried different numbers between 15 and 30, and selected the number that maximized the corresponding silhouette coefficient.

We calculated the final ARI and NMI values of scAce given the three different algorithms for cluster initialization. The difference in ARI values was between 0.001 and 0.069 across the seven real datasets, and the difference in NMI values was between 0.001 and 0.059 (Supplementary Fig. S11). Our results show that scAce is robust to the selection of the initial clustering algorithm. In fact, regardless of which initial clustering algorithm is used, as long as the initial clusters are of high purity, scAce is expected to merge highly similar small clusters into larger ones in the adaptive merging process. In addition to its robustness to initial clustering algorithms, we also observed that scAce was robust to the resolution parameter in the Leiden algorithm when its value was between 1.4 and 2.0 (Supplementary Table S5).

### 3.4 scAce enhances the performance of existing clustering methods

**Figure 4.**
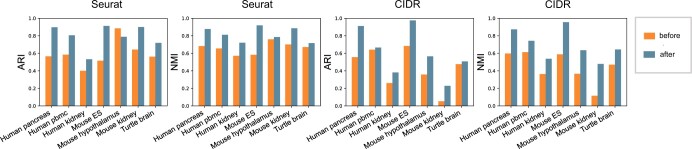
ARI and NMI values of clustering results before and after scAce’s clustering enhancement for Seurat and CIDR.

We provide a clustering enhancement option in the scAce method, which allows it to start from cluster labels inferred by another scRNA-seq clustering method and use its cluster splitting, VAE network update, and cluster merging steps to adaptively improve the clustering results (see Section 2 and Supplementary Methods). To evaluate the effectiveness of this option, we implemented clustering enhancement by applying scAce with initial cluster labels obtained by Seurat or CIDR, both of which do not utilize neural networks or cluster merging approaches. Our results show that, given initial cluster labels from either Seurat or CIDR, scAce obviously improved the final clustering accuracy on most real datasets, in terms of both ARI and NMI scores (Fig. 4).

We visualized the cell embeddings and cluster labels obtained by scAce right after pretraining and cluster initialization, and the final embeddings and labels after clustering enhancement, given initial results from Seurat (Supplementary Fig. S12) and CIDR (Supplementary Fig. S13), respectively. Compared with the original clustering results of Seurat and CIDR, scAce obtained smaller and purer clusters after its initialization (Supplementary Methods). Supplementary Figures S12B and S13B show the cell embeddings and cluster labels given scAce’s final output, which demonstrates scAce’s ability to enhance cluster assignment and learning of the latent space through adaptive cluster merging.

### 3.5 Computational time and memory usage

We measured the running time and maximum memory usage of scAce and the other nine methods on the seven real datasets (Supplementary Fig. S14). In the experiments, CIDR, SCCAF, and Seurat only needed to use CPUs, and the other seven methods needed GPUs. All experiments used a single core. The average running time of scAce ranked fourth, and Seurat and ADClust were the fastest. Although scAce was slightly slower than Seurat and ADClust, it was on average faster than the other deep-learning-based methods (scGMAAE, scVI, scDeepCluster, graph-sc, and DESC) (Supplementary Fig. S14A). As for the memory usage, the maximum memory usage of scAce was higher than most alternative methods, but was on the same magnitude as that of other deep-learning-based methods (Supplementary Fig. S14B).

## 4 Discussion

In this article, we propose the scAce method for unsupervised clustering analysis of single cells using scRNA-seq data. Using a variational autoencoder network that adaptively learns both low-dimensional cell representations and cluster assignment, scAce allows for accurate clustering of cells without the need to predetermine the cluster number or other parameters indicating preferences on cluster resolution. We evaluated the performance of scAce in comparison with nine alternative clustering methods for scRNA-seq data based on both simulated and real datasets. Our results suggest that scAce outperforms existing state-of-the-art methods in terms of both clustering accuracy and robustness. Based on the clustering enhancement option of scAce, it is also possible to further improve the accuracy of an existing clustering assignment generated by other methods.

Compared with existing deep embedding clustering algorithms that use traditional autoencoders to obtain low-dimensional representations, the VAE network used by scAce obtains better low-dimensional embeddings of single cells and therefore improves clustering results by enforcing a continuous and smooth latent space representation of the gene expression data. Another feature of scAce that contributes to its improved clustering performance is its ability to adaptively update the cluster assignments in a deep embedding framework. scAce starts with relatively large number of clusters which have a high purity and iteratively merges similar clusters together using the proposed adaptive cluster merging approach. This approach takes advantage of the trained VAE network and simultaneously achieves network update and cluster update to improve both cell embeddings and cluster assignments.

When summarizing our computational results, we noticed that even though scAce successfully identified the rare cell type (with a cell proportion of 4%) in the simulated data, it did not always identify the rare cell types in the real datasets. Actually, none of the 10 methods evaluated in this work was able to consistently identify rare cell types that only accounted for between 0.027% and 1.498% of cells. In a recent benchmark study of 14 clustering methods, it was also reported that most methods significantly underestimated the true cell type numbers when the proportion of cells in rare cell types was around 2% (Yu *et al.* 2022). Since this is a systematic challenge, a future direction is to investigate how to further improve the identification of rare cell types. One possible solution is to first evaluate the heterogeneity of inferred cell clusters (Li 2022, Liu *et al.* 2020), and then perform another round of clustering analysis just using the cell clusters appearing to be mixtures of multiple cell types. When the focus is to discover rare cell types, an alternative solution is to modify the adaptive cluster merging process in scAce and use a more stringent merging criterion. Another future direction to consider is how to extend scAce to account for the cell type hierarchy. There have been a few methods which aim to use a tree structure to recover hierarchical relationships among cell types or account for this factor when evaluating clustering results (Wu and Wu 2020, Peng *et al.* 2021). As scAce is based on an adaptive cluster merging approach, it would be possible to learn cluster hierarchy from the merging orders.

Although scAce has been developed as a clustering method for scRNA-seq data, we believe that its VAE framework and adaptive cluster merging approach can be extended to model additional types of data collected from other technologies, such as scATAC-seq and spatial transcriptomics (Lei *et al.* 2021). The framework can also be modified for noncount data by changing the output format of the decoder and the corresponding loss functions. Given the pivotal role of clustering analysis in single-cell studies and many other scientific problems, we anticipate scAce to be a useful method for discovering meaningful clusters in high-dimensional data.

## Supplementary Material

btad546_Supplementary_Data

## Data Availability

scAce is implemented as a Python package, which is freely available from its GitHub repository https://github.com/sldyns/scAce. The accession numbers of all real data used in this work are described in Supplementary Table S2. The data and code used to reproduce the analyses are available at https://github.com/sldyns/scAce/tree/main/reproducibility.

## References

[btad546-B1] Adam M , Potter AS, Potter SS et al Psychrophilic proteases dramatically reduce single-cell RNA-seq artifacts: a molecular atlas of kidney development. Development 2017;144:3625–32. 28851704 10.1242/dev.151142 PMC5665481

[btad546-B2] Baron M , Veres A, Wolock SL et al A single-cell transcriptomic map of the human and mouse pancreas reveals inter-and intra-cell population structure. Cell Syst 2016;3:346–60.e4. 27667365 10.1016/j.cels.2016.08.011 PMC5228327

[btad546-B3] Blondel VD , Guillaume J-L, Lambiotte R et al Fast unfolding of communities in large networks. J Stat Mech 2008;2008:P10008.

[btad546-B4] Chen R , Wu X, Jiang L et al Single-cell RNA-seq reveals hypothalamic cell diversity. Cell Rep 2017;18:3227–41. 28355573 10.1016/j.celrep.2017.03.004 PMC5782816

[btad546-B5] Choi Y , Li R, Quon G et al sivae: interpretable deep generative models for single-cell transcriptomes. Genome Biol 2023;24:29. 36803416 10.1186/s13059-023-02850-y PMC9940350

[btad546-B6] Ciortan M , Defrance M. GNN-based embedding for clustering scRNA-seq data. Bioinformatics 2022;38:1037–44. 34850828 10.1093/bioinformatics/btab787

[btad546-B7] Deng Y , Bao F, Dai Q et al Scalable analysis of cell-type composition from single-cell transcriptomics using deep recurrent learning. Nat Methods 2019;16:311–4. 30886411 10.1038/s41592-019-0353-7 PMC6774994

[btad546-B8] Eraslan G , Simon LM, Mircea M et al Single-cell RNA-seq denoising using a deep count autoencoder. Nat Commun 2019;10:390. 30674886 10.1038/s41467-018-07931-2 PMC6344535

[btad546-B9] Grønbech CH , Vording MF, Timshel PN et al scvae: variational auto-encoders for single-cell gene expression data. Bioinformatics 2020;36:4415–22. 32415966 10.1093/bioinformatics/btaa293

[btad546-B10] Guo X , Gao L, Liu X et al Improved deep embedded clustering with local structure preservation. In: *Proceedings of 26th International Joint Conference on Artificial Intelligence, IJCAI*, Melbourne, Australia, 2017, 1753–9.

[btad546-B11] Higgins I , Matthey L, Pal A et al beta-vae: Learning basic visual concepts with a constrained variational framework. In: 5**th* International Conference on Learning Representations*, *Toulon, France*, 2017.

[btad546-B12] Kingma DP , Welling M. Auto-encoding variational Bayes. arXiv prerint, arXiv:1312.6114, 2022, preprint: not peer reviewed.

[btad546-B13] Kiselev VY , Andrews TS, Hemberg M et al Challenges in unsupervised clustering of single-cell RNA-seq data. Nat Rev Genet 2019;20:273–82. 30617341 10.1038/s41576-018-0088-9

[btad546-B14] Kiselev VY , Kirschner K, Schaub MT et al Sc3: consensus clustering of single-cell RNA-seq data. Nat Methods 2017;14:483–6. 28346451 10.1038/nmeth.4236 PMC5410170

[btad546-B15] Klein AM , Mazutis L, Akartuna I et al Droplet barcoding for single-cell transcriptomics applied to embryonic stem cells. Cell 2015;161:1187–201. 26000487 10.1016/j.cell.2015.04.044 PMC4441768

[btad546-B16] Lei J , Jiang T, Wu K et al Robust k-means algorithm with automatically splitting and merging clusters and its applications for surveillance data. Multimed Tools Appl 2016;75:12043–59.

[btad546-B17] Lei Y , Tang R, Xu J et al Applications of single-cell sequencing in cancer research: progress and perspectives. J Hematol Oncol 2021;14:91. 34108022 10.1186/s13045-021-01105-2 PMC8190846

[btad546-B18] Li WV. Phitest for analyzing the homogeneity of single-cell populations. Bioinformatics 2022;38:2639–41. 35238346 10.1093/bioinformatics/btac130 PMC9048696

[btad546-B19] Li X , Wang K, Lyu Y et al Deep learning enables accurate clustering with batch effect removal in single-cell RNA-seq analysis. Nat Commun 2020;11:2338. 32393754 10.1038/s41467-020-15851-3 PMC7214470

[btad546-B20] Lin P , Troup M, Ho JWK et al CIDR: ultrafast and accurate clustering through imputation for single-cell RNA-seq data. Genome Biol 2017;18:59. 28351406 10.1186/s13059-017-1188-0 PMC5371246

[btad546-B21] Liu B , Li C, Li Z et al An entropy-based metric for assessing the purity of single cell populations. Nat Commun 2020;11:3155. 32572028 10.1038/s41467-020-16904-3 PMC7308400

[btad546-B22] Lopez R , Regier J, Cole MB et al Deep generative modeling for single-cell transcriptomics. Nat Methods 2018;15:1053–8. 30504886 10.1038/s41592-018-0229-2 PMC6289068

[btad546-B23] Miao Z , Moreno P, Huang N et al Putative cell type discovery from single-cell gene expression data. Nat Methods 2020;17:621–8. 32424270 10.1038/s41592-020-0825-9

[btad546-B24] Peng M , Wamsley B, Elkins AG et al Cell type hierarchy reconstruction via reconciliation of multi-resolution cluster tree. Nucleic Acids Res 2021;49:e91. 34125905 10.1093/nar/gkab481 PMC8450107

[btad546-B25] Petegrosso R , Li Z, Kuang R. Machine learning and statistical methods for clustering single-cell rna-sequencing data. Brief Bioinform 2020;21:1209–22. 31243426 10.1093/bib/bbz063

[btad546-B26] Qi R , Ma A, Ma Q et al Clustering and classification methods for single-cell RNA-sequencing data. Brief Bioinform 2020;21:1196–208. 31271412 10.1093/bib/bbz062 PMC7444317

[btad546-B27] Satija R , Farrell JA, Gennert D et al Spatial reconstruction of single-cell gene expression data. Nat Biotechnol 2015;33:495–502. 25867923 10.1038/nbt.3192 PMC4430369

[btad546-B28] Sheng J , Li WV. Selecting gene features for unsupervised analysis of single-cell gene expression data. Brief Bioinform 2021;22:bbab295. 34351383 10.1093/bib/bbab295 PMC8574996

[btad546-B29] Sun T , Song D, Li WV et al scdesign2: a transparent simulator that generates high-fidelity single-cell gene expression count data with gene correlations captured. Genome Biol 2021;22:163. 34034771 10.1186/s13059-021-02367-2 PMC8147071

[btad546-B30] Tian T , Wan J, Song Q et al Clustering single-cell RNA-seq data with a model-based deep learning approach. Nat Mach Intell 2019;1:191–8.

[btad546-B31] Tian T , Zhang J, Lin X et al Model-based deep embedding for constrained clustering analysis of single cell RNA-seq data. Nat Commun 2021;12:1873. 33767149 10.1038/s41467-021-22008-3 PMC7994574

[btad546-B32] Tosches MA , Yamawaki TM, Naumann RK et al Evolution of pallium, hippocampus, and cortical cell types revealed by single-cell transcriptomics in reptiles. Science 2018;360:881–8. 29724907 10.1126/science.aar4237

[btad546-B33] Traag VA , Waltman L, van Eck NJ et al From louvain to leiden: guaranteeing well-connected communities. Sci Rep 2019;9:5233. 30914743 10.1038/s41598-019-41695-z PMC6435756

[btad546-B34] Wang D , Gu J. Vasc: dimension reduction and visualization of single-cell RNA-seq data by deep variational autoencoder. Genomics Proteomics Bioinf 2018;16:320–31. 10.1016/j.gpb.2018.08.003 PMC6364131 30576740

[btad546-B35] Wang H-Y , Zhao J-P, Zheng C-H, et al scgmaae: Gaussian mixture adversarial autoencoders for diversification analysis of scRNA-seq data. Brief Bioinform 2023;24:bbac585. 36592058 10.1093/bib/bbac585

[btad546-B36] Ward JH Jr . Hierarchical grouping to optimize an objective function. J Am Stat Assoc 1963;58:236–44.

[btad546-B37] Wolf FA , Angerer P, Theis FJ et al Scanpy: large-scale single-cell gene expression data analysis. Genome Biol 2018;19:15. 29409532 10.1186/s13059-017-1382-0 PMC5802054

[btad546-B38] Wu Z , Wu H. Accounting for cell type hierarchy in evaluating single cell RNA-seq clustering. Genome Biol 2020;21:123. 32450895 10.1186/s13059-020-02027-x PMC7249323

[btad546-B39] Xie J , Girshick R, Farhadi A. Unsupervised deep embedding for clustering analysis. In: *International Conference on Machine Learning.* New York, United States: PMLR 2016, 478–87.

[btad546-B40] Young MD , Mitchell TJ, Vieira Braga FA et al Single-cell transcriptomes from human kidneys reveal the cellular identity of renal tumors. Science 2018;361:594–9. 30093597 10.1126/science.aat1699 PMC6104812

[btad546-B41] Yu L , Cao Y, Yang JYH et al Benchmarking clustering algorithms on estimating the number of cell types from single-cell RNA-sequencing data. Genome Biol 2022;23:49. 35135612 10.1186/s13059-022-02622-0 PMC8822786

[btad546-B42] Zeng Y et al A parameter-free deep embedded clustering method for single-cell RNA-seq data. Brief Bioinform 2022;23:bbac172. 35524494 10.1093/bib/bbac172

[btad546-B43] Zheng GXY , Terry JM, Belgrader P et al Massively parallel digital transcriptional profiling of single cells. Nat Commun 2017;8:14049. 28091601 10.1038/ncomms14049 PMC5241818

