# Supplementary material for: scAce: an adaptive embedding and clustering method for single-cell gene expression data
Source: Bioinformatics. 2023 Sep 6;39(9):btad546. doi: 10.1093/bioinformatics/btad546 (PMC10500084; doi:10.1093/bioinformatics/btad546)
Supplement: btad546_Supplementary_Data [file btad546_supplementary_data.pdf]

Supplementary materials for "scAce: an adaptive clustering method for scRNA-seq data based on cluster merging"

Supplementary Tables

Table S1: ARI values given different values of λ on the eight datasets. To evaluate the impact λ's value, we tried different values in 100, 10, 1, 0.1 and 0.01, while controlling the other parameters to remain unchanged, and conducted experiments on the simulated dataset and seven real datasets.

Dataset	100	10	1 (default)	0.1	0.01
Simulation	0.81	0.81	**1.00**	0.78	0.78
Human pancreas	0.89	**0.95**	0.89	0.87	0.51
Human PBMC	0.76	0.76	**0.81**	0.65	0.39
Human kidney	0.16	0.62	**0.65**	0.40	0.38
Mouse ES	0.90	**0.91**	0.90	0.79	0.77
Mouse hypothalamus	0.77	0.71	**0.84**	0.73	0.60
Mouse kidney	0.82	0.72	**0.93**	0.55	0.25
Turtle brain	0.71	**0.83**	**0.83**	0.50	0.33
Average	0.72	0.79	**0.86**	0.66	0.50

Table S2: ARI values given different values of β during adaptive cluster merging on the eight datasets. In the pre-training stage, the main goal of scAce is to obtain an initialized VAE network, so we set β to a relative small value, $0.01 \times m$. At the adaptive cluster merging stage, the value of β directly affect the final clustering performance, so we focused on evaluating its impact in this stage. We evaluated β's value in $0.01 \times m$, $0.007 \times m$, $0.004 \times m$ and $0.001 \times m$, while controlling the other parameters to remain unchanged, and conducted experiments on the simulated dataset and seven real datasets.

Dataset	$0.01 \times m$ (default)	$0.007 \times m$	$0.004 \times m$	$0.001 \times m$
Simulation	**1.00**	**1.00**	0.81	0.81
Human pancreas	**0.89**	**0.89**	**0.89**	**0.89**
Human PBMC	**0.81**	0.79	0.79	**0.81**
Human kidney	0.65	0.66	**0.75**	0.64
Mouse ES	0.90	0.84	**0.91**	0.90
Mouse hypothalamus	**0.84**	**0.84**	**0.84**	**0.84**
Mouse kidney	**0.93**	0.92	0.92	0.83
Turtle brain	**0.83**	0.81	0.76	0.76
Average	**0.86**	0.84	0.84	0.81

Table S3: A summary of the ten clustering methods for scRNA-seq data.

Name	Methods	Cluster number required	Programming language
ADClust	Autoencoder + Cluster merging	×	Python
CIDR	Imputation + Hierarchical clustering	×	R
DESC	Autoencoder	×	Python
graph-sc	Graph autoencoder + K-means	✓	Python
SCCAF	Self-projection + Cluster merging	×	Python
scDeepCluster	Autoencoder	✓	Python
scGMAAE	VAE + Spectral clustering	✓	Python
scVI	VAE	×	Python
Seurat	Shared-nearest-neighbor-based clustering	×	R
scAce	VAE + Cluster merging	×	Python

Table S4: A summary of the seven real scRNA-seq datasets. For the Human pancreas dataset, the cell type annotations were obtained based on expression of unique transcripts and with reference to literature, after an iterative hierarchical clustering analysis. For the Human PBMC dataset, the cell type annotations were determined by the FACS sorting. For Human kidney dataset, the cell type annotations were obtained based on marker genes curated from literature, after graph-based clustering. For the Mouse ES dataset, the cell labels were directly determined by time points after experiment treatment. For the Mouse hypothalamus dataset, the cell type annotations were obtained based on known marker genes, after density-based clustering. For the Mouse kidney and Turtle brain datasets, the cell type annotations were obtained based on known marker genes, after graph-based clustering.

Datasets	No. of cells	No. of genes	No. of cell types	Source
Human pancreas	3605	20125	14	GEO accession number GSM2230758
Human PBMC	4271	16653	8	GEO accession number GSE96583
Human kidney	5685	25215	11	EGA accession number EGAS00001002171
Mouse ES	2717	24047	4	GEO accession number GSE65525
Mouse hypothalamus	12089	23284	46	GEO accession number GSE87544
Mouse kidney	3660	23797	8	GEO accession number GSE94333
Turtle brain	18664	23500	15	NCBI accession number PRJNA408230

Table S5: ARI values of scAce given different resolution parameters used in the cluster initialization. To investigate the impact of cluster initialization on the performance of scAce, we set the resolution parameter in the Leiden algorithm to values in 0.05, 0.2, 0.5, 0.8, 1.1, 1.4, 1.7, and 2.0, and calculated the ARI values of the corresponding final clustering results.

Dataset	0.05	0.2	0.5	0.8	1.1	1.4	1.7	2.0 (default)
Human pancreas	0.90	0.95	0.90	0.89	0.95	**0.96**	0.89	0.89
Human PBMC	0.42	0.69	0.76	0.67	0.66	0.76	**0.81**	**0.81**
Human kidney	0.52	0.59	0.53	0.59	0.59	0.58	**0.71**	0.64
Mouse ES	0.86	**0.90**	**0.90**	**0.90**	**0.90**	**0.90**	**0.90**	**0.90**
Mouse hypothalamus	0.76	0.83	0.83	0.83	0.83	0.83	0.84	**0.86**
Mouse kidney	0.77	0.91	0.92	0.92	**0.93**	**0.93**	0.91	**0.93**
Turtle brain	0.64	0.60	0.78	0.72	0.57	**0.79**	0.64	0.71
Average	0.70	0.78	0.77	0.78	0.78	**0.82**	0.81	**0.82**

Supplementary Figures

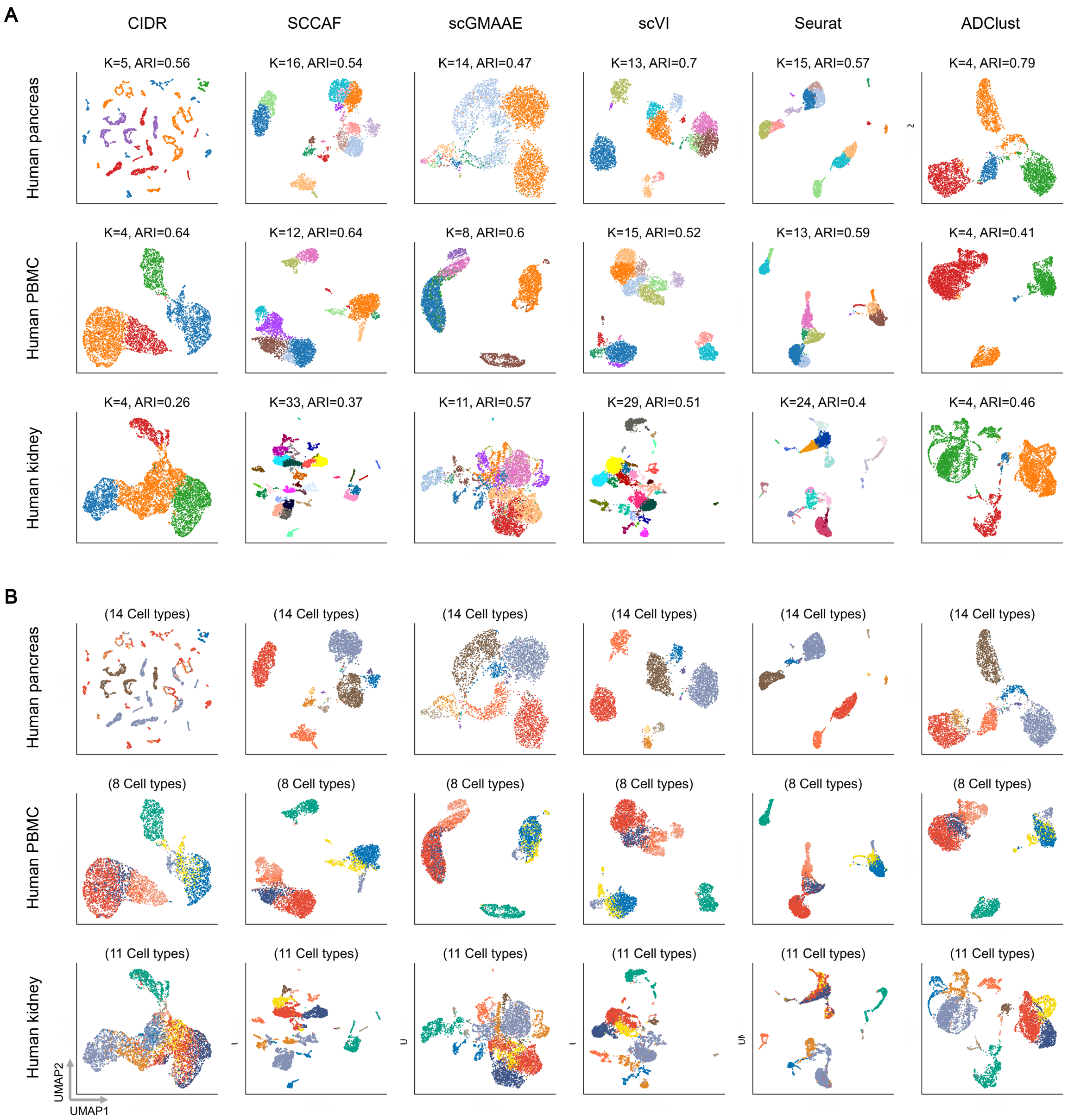

Figure S1: UMAP plots of the cell embeddings produced by six methods (CIDR, SCCAF, scGMAAE, scVI, Seurat, and ADClust) on the three human datasets. **(A)** The cells are colored by inferred cluster labels. **(B)** The cells are colored by true cell types.

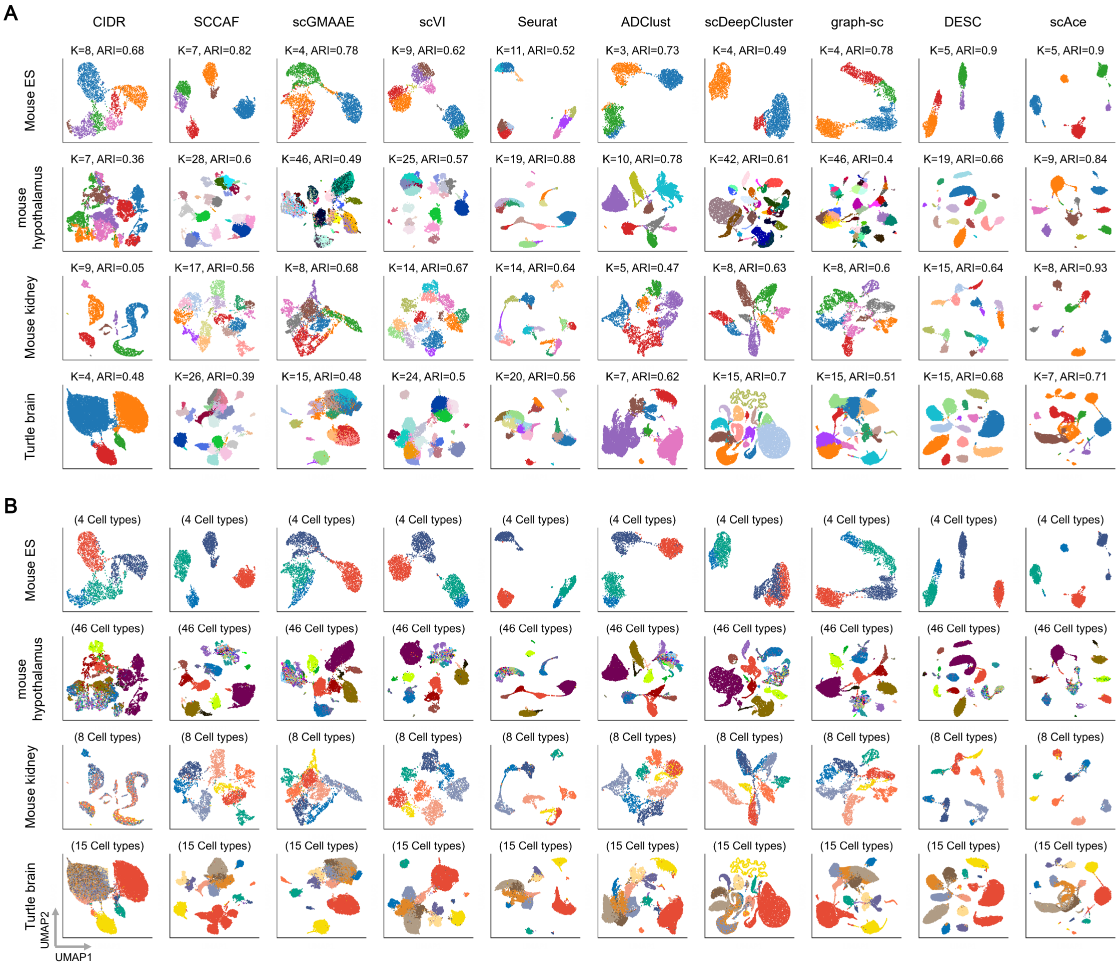

Figure S2: UMAP plots of the cell embeddings produced by the ten methods on the mouse and turtle datasets. **(A)** The cells are colored by inferred cluster labels. **(B)** The cells are colored by true cell types.

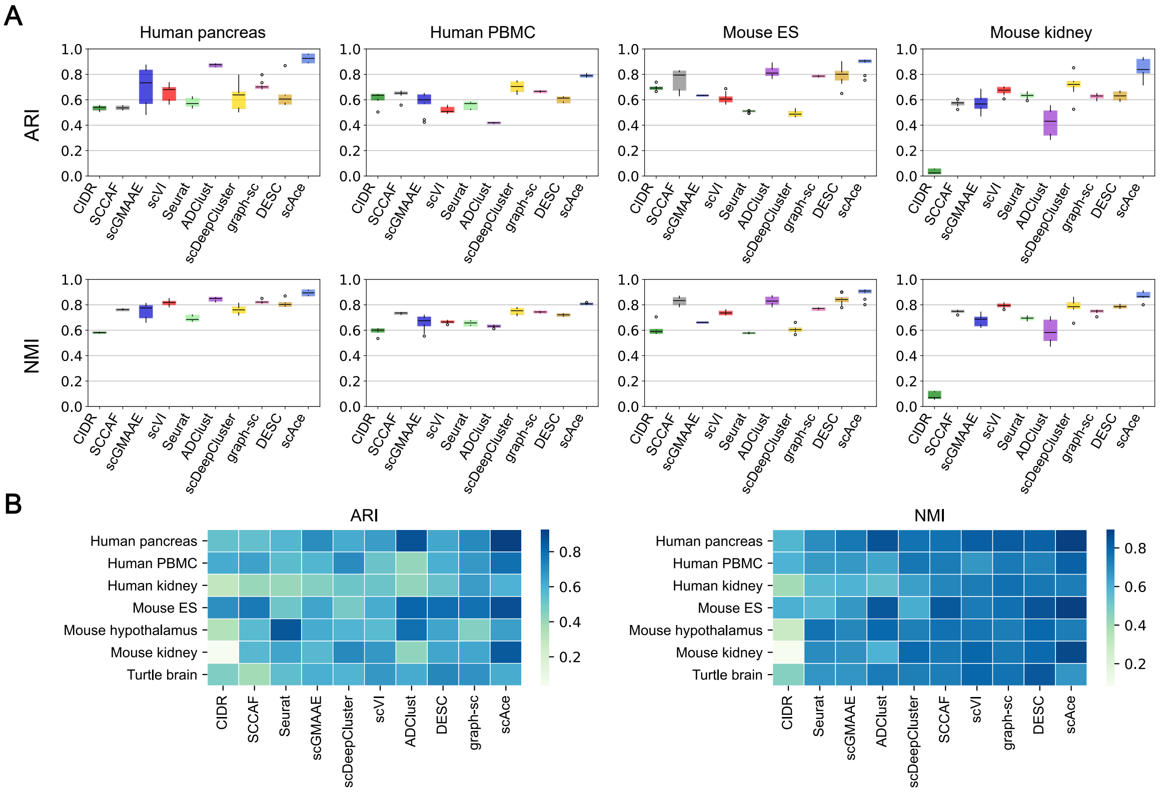

Figure S3: Comparison of the ten clustering methods on subsets of real datasets. **(A)** Boxplots of ARI and NMI values obtained by applying the ten clustering methods to randomly selected subsamples of the full datasets. **(B)** Mean values of ARI and NMI obtained from the 10 repeated experiments for each of the ten methods on the seven real datasets. The methods are ordered based on the mean ARI/NMI values.

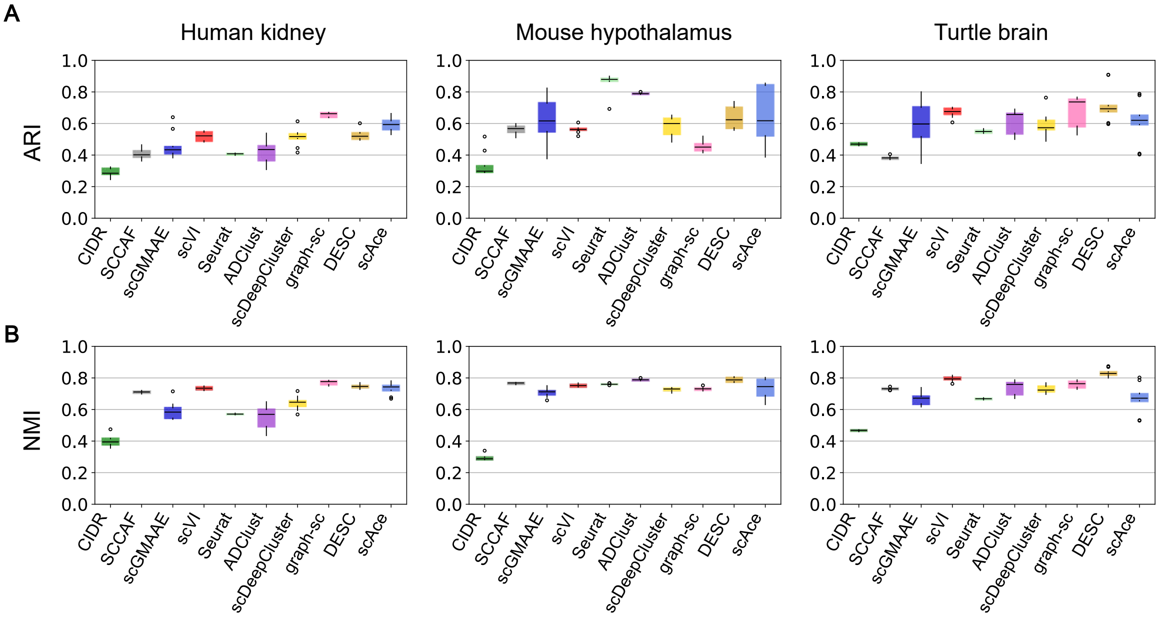

Figure S4: Comparison of the clustering methods on three real scRNA-seq datasets (Human kidney, Mouse hypothalamus, and Turtle brain) by applying the ten clustering methods to randomly selected subsamples of the full datasets. **(A)** Boxplots of ARI values. **(B)** Boxplots of NMI values.

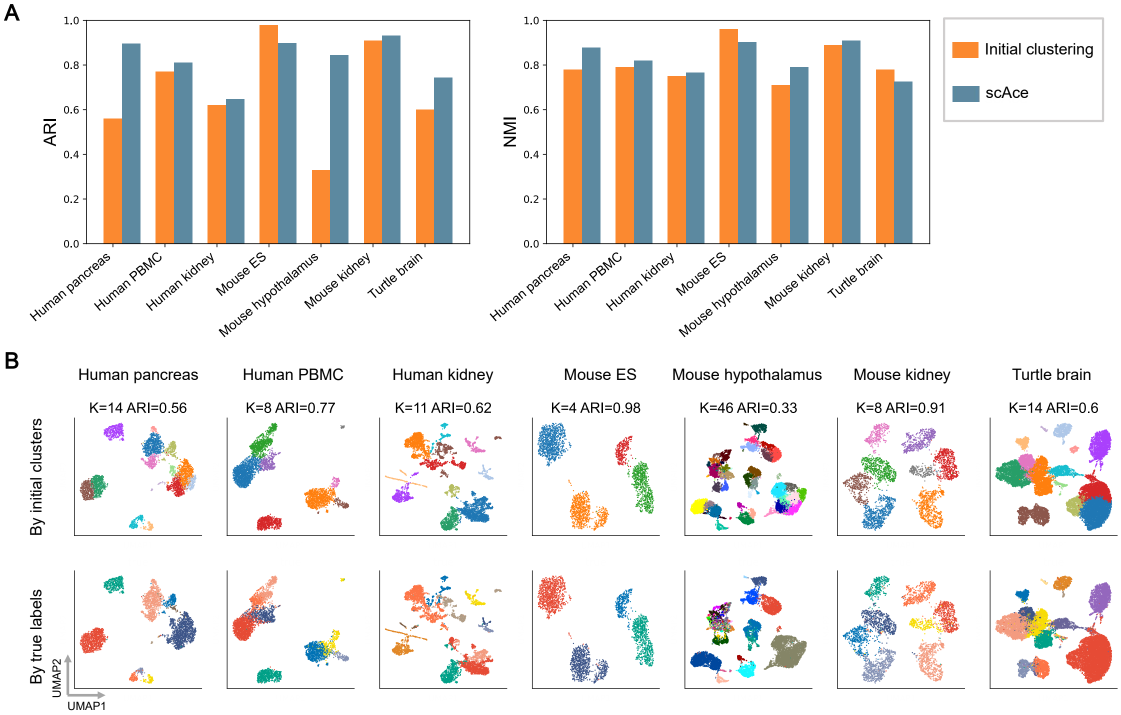

Figure S5: Comparison between results of scAce and cluster initialization. **(A)** ARI and NMI values of scAce's clustering results and initial clustering results obtained by setting the resolution parameter in the Leiden algorithm such that the initial cluster number was the same as the true cell type number. **(B)** UMAP plots of initial clusters obtained by setting the resolution parameter in the Leiden algorithm such that the initial cluster number was the same as the true cell type number.

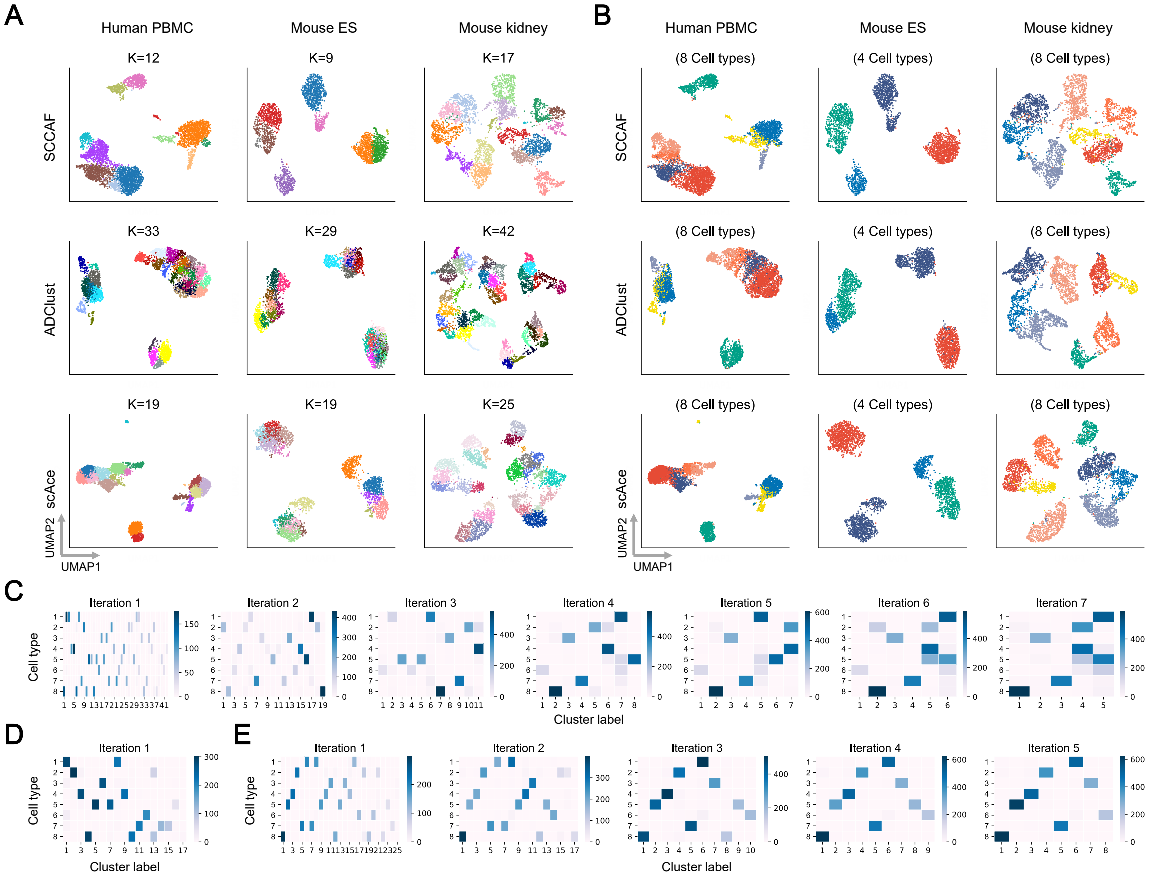

Figure S6: Comparison of SCCAF, ADClust, and scAce in the cluster merging process. **(A)** UMAP plots of the initial clustering results of SCCAF, ADClust, and scAce on Human PBMC, Mouse ES, and Mouse kidney datasets. The cells are colored by inferred cluster labels. The estimated numbers of initial clusters (K) of each method are labeled on top of the corresponding plots. **(B)** UMAP plots same as shown in **A** but colored by true cell types. **(C-E)** Heatmaps of ADClust (**C**), SCCAF (**D**), and scAce (**E**) showing the degree of similarity between the inferred clusters and true cell types (on the Mouse kidney dataset). The horizontal axis represents the inferred cell clusters and the vertical axis represents the true cell types. The color represents the number of cells of a cell type assigned to a inferred cluster.

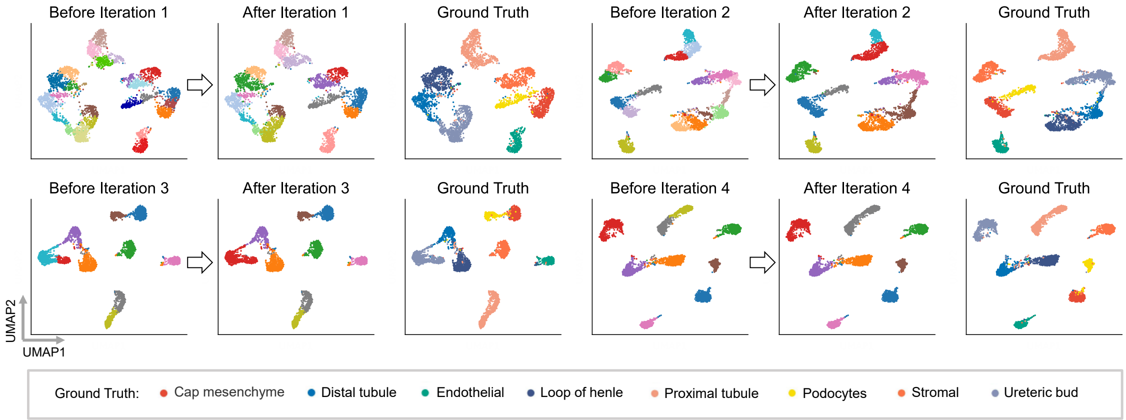

Figure S7: UMAP plots of clustering results and ground truth cell types before and after each iteration of scAce's adaptive merging (Mouse kidney dataset).

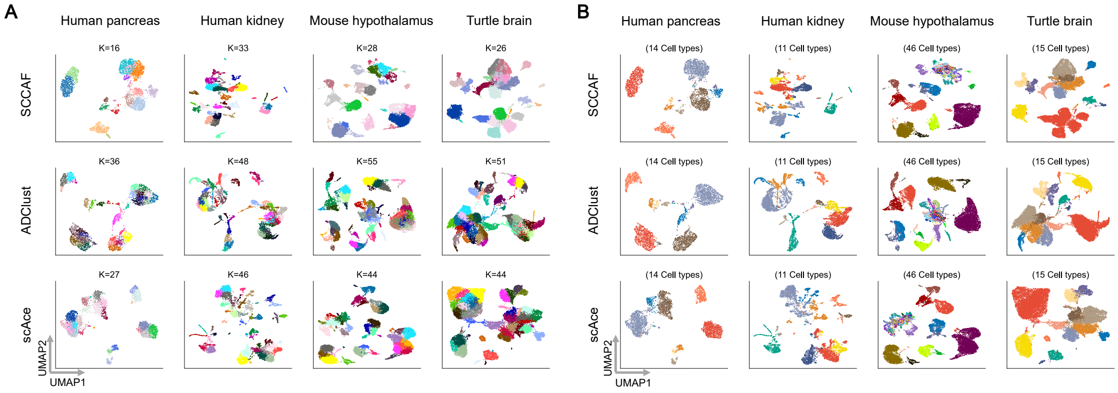

Figure S8: UMAP plots of the initial clustering results of ADClust, SCCAF, and scAce on the Human pancreas, Human kidney, Mouse hypothalamus and Turtle brain datasets. **(A)** Each point represents a cell, and each color represents a predicted cluster. The estimated numbers of initial clusters (K) of each method are labeled on top of the corresponding plots. **(B)** Each point represents a cell, and each color represents a ground truth cell type.

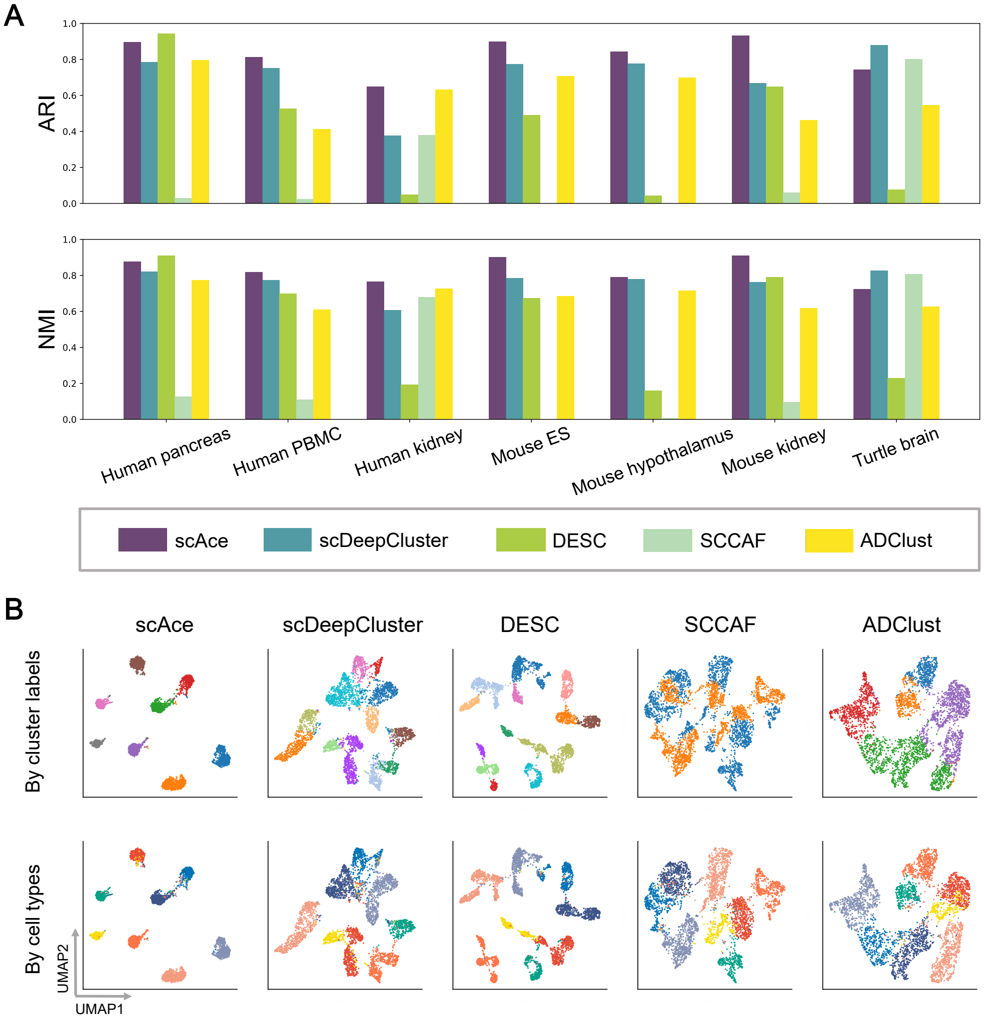

Figure S9: Comparison of cluster merging results based on different methods (scAce, scDeepCluster, DESC, SCCAF, and ADClust). **(A)** ARI and NMI values of scAce and the other four revised methods. **(B)** UMAP plots for the Mouse kidney dataset based on the five methods. The cells are colored by the inferred cluster labels. **(C)** UMAP plots same as shown in **B** but colored by the true cell types.

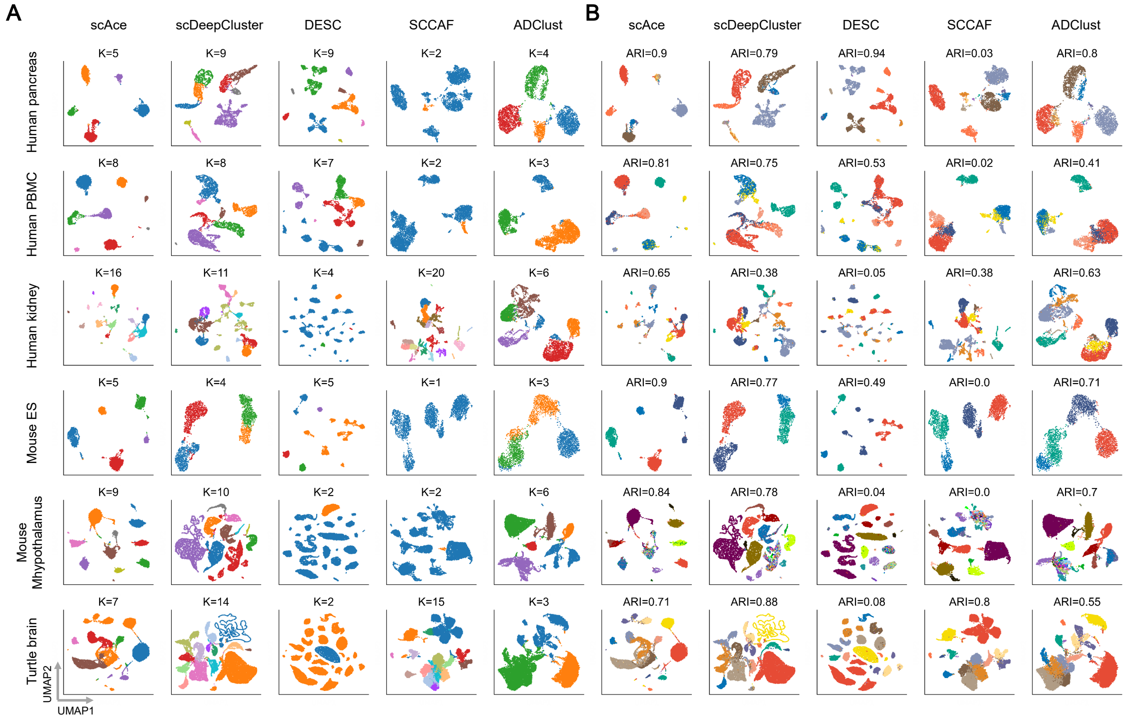

Figure S10: UMAP plots of the cell embeddings produced by scAce and the other four revised methods. **(A)** The cells are colored by inferred cluster labels. **(B)** The cells are colored by true cell types.

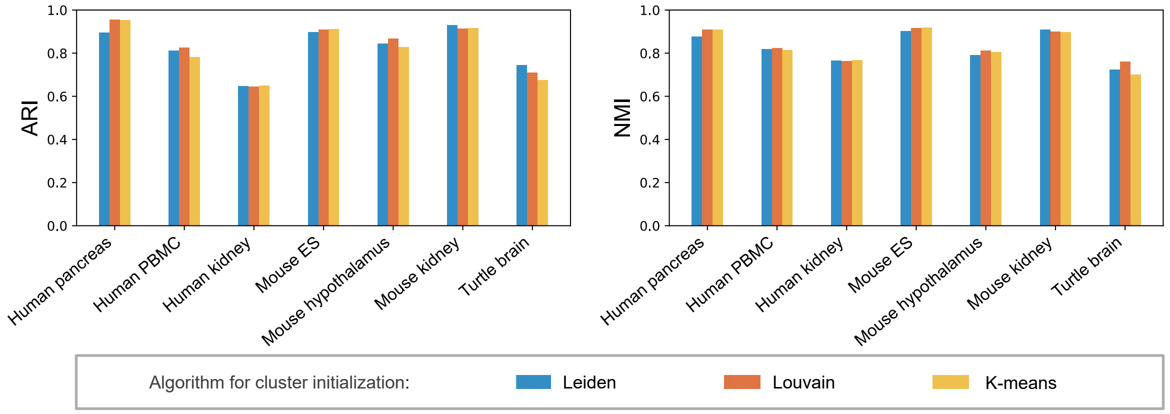

Figure S11: Comparison of scAce performance based on three different cluster initialization algorithms.

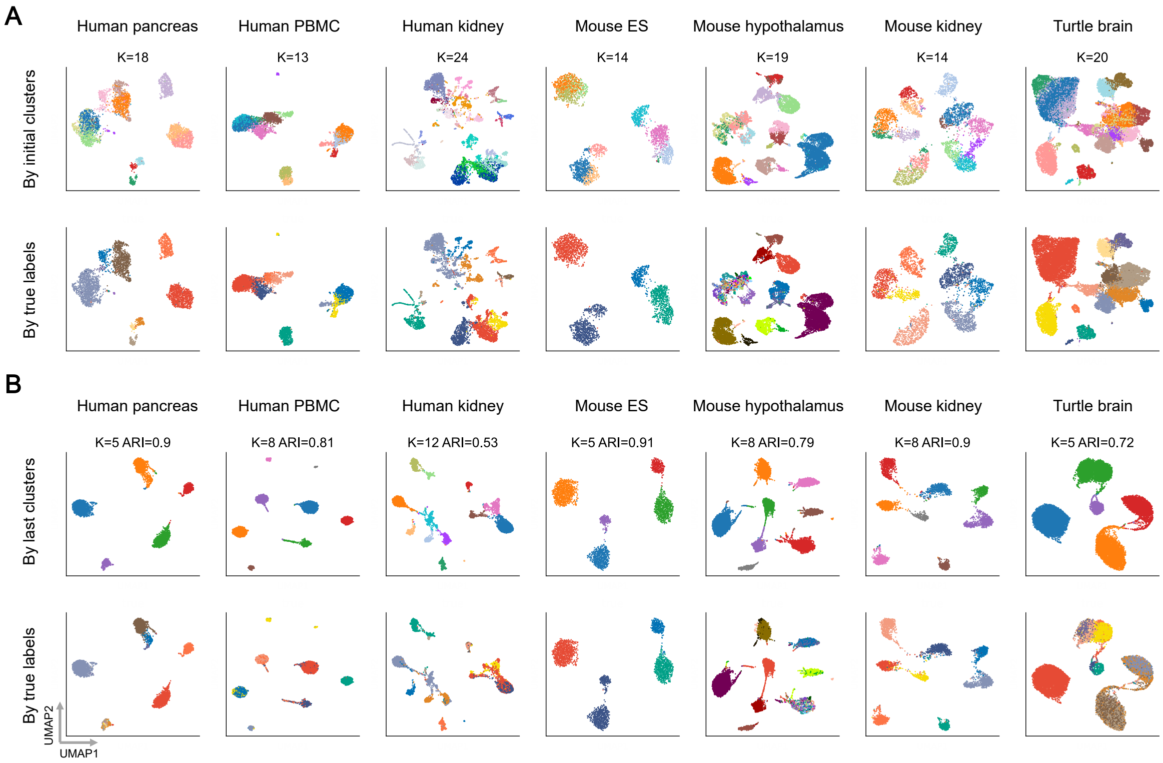

Figure S12: UMAP plots of scAce's clustering enhancement based on Seurat. **(A)** UMAP plots of initialized clusters obtained by scAce based on Seurat's clustering results. The two rows of plots are colored by initial scAce cluster labels and true cell types, respectively. **(B)** UMAP plots of the final clustering results after scAce performed clustering enhancement for Seurat. The two rows of plots are colored by final scAce cluster labels and true cell types, respectively.

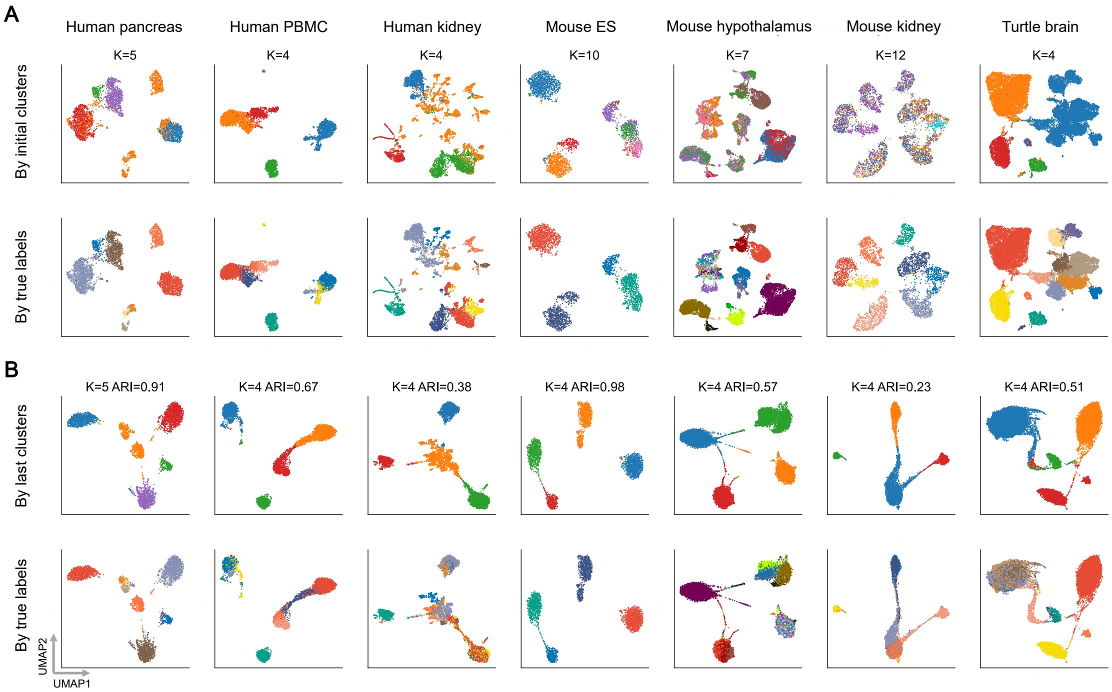

Figure S13: The effect of clustering enhancement on CIDR. **(A)** UMAP plots of initialized clusters obtained by scAce based on CIDR's clustering results. **(B)** UMAP plots of the clustering results after scAce performed clustering enhancement for CIDR.

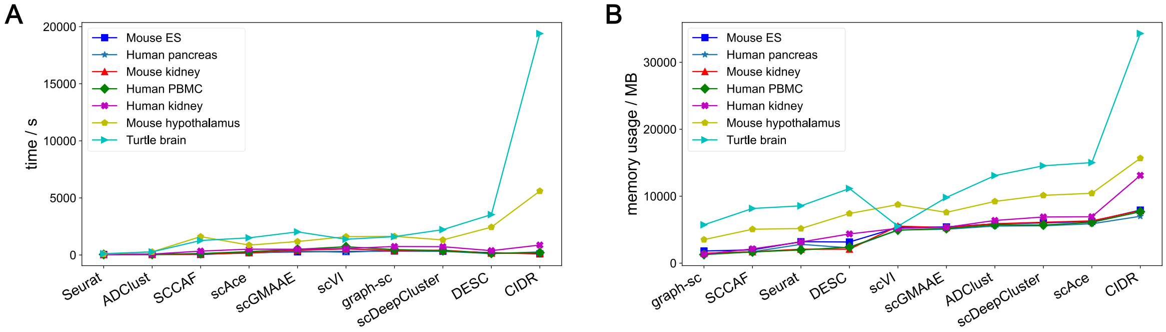

Figure S14: Comparison of running time and memory usage. **(A)** Comparison of running time for the ten methods on the seven real datasets. **(B)** Comparison of memory usage for the ten methods on the seven real datasets. The methods are ordered based on the average running time or memory usage across datasets.

Supplementary Methods

Data pre-processing

For all scRNA-seq datasets used, scAce pre-processes the raw count matrix using Scanpy [?]. The pre-processing steps are as follows. First, genes expressed in fewer than 3 cells and cells with fewer than 200 expressed genes are filtered out. The remaining count matrix is denoted as $X = [x_{ij}] \in \mathbb{R}^{m\times n}$. Second, the read counts in each cell are normalized by the library size factor of that cell. The library size factor is defined as the total count in a cell divided by the median total counts across all cells. Third, the normalized counts are log-transformed and then converted to z-scores, such that the expression level of every gene has zero mean and unit variance across all cells. The processed gene expression matrix is denoted as $\tilde{X} \in \mathbb{R}^{m\times n}$.

Derivation of the VAE loss function

For sample X, our goal is to use only X to learn the joint distribution of data X and the hidden variable Z through an unsupervised deep generative model such that the observed data X can be generated through Z using the model $p(X|Z)$. Assuming that the model is controlled by the parameter θ, our goal can then be expressed as maximizing the expected log-likelihood value of the observed data X over the entire distribution of the hidden variable Z as follows [1]:

$$\max_{\theta} \mathbb{E}_{p_\theta(Z)}\left[\log p_\theta(X|Z)\right]. \tag{S1}$$

Since we do not know the exact form of the true $p(Z)$, we define the variational distribution $q_\phi(Z|X)$ to approximate $p(Z)$. The KL divergence is used to measure how close the two distributions are to one another; a smaller KL divergence denotes a greater similarity between the two distributions.

$$\max \mathbb{E}_{q_\phi(Z|X)}\left[\log p_\theta(X|Z)\right] \quad \text{s.t.} \quad \mathrm{D_{KL}}(q_\phi(Z|X)\|p(Z)) \le \epsilon. \tag{S2}$$

Converting them to the Lagrangian form, we obtain the following objective function:

$$\mathscr{F} = \mathbb{E}_{q_\phi(Z|X)}\left[\log p_\theta(X|Z)\right] - \beta(\mathrm{D_{KL}}(q_\phi(Z|X)\|p(Z)) - \epsilon), \tag{S3}$$

where $\beta \ge 1$ is the regularisation factor and the model becomes the conventional VAE model when $\beta = 1$. Since a small KL divergence term will lead to poor reconstructability of the model, the KL divergence term is enhanced by setting the coefficient β to improve the reconstructability of the model. Ignoring the fixed constants, the objective function can be written as:

$$\mathscr{F} = \mathbb{E}_{q_\phi(Z|X)}\left[\log p_\theta(X|Z)\right] - \beta\mathrm{D_{KL}}(q_\phi(Z|X)\|p(Z)). \tag{S4}$$

To better characterize the scRNA-seq count data, we use the ZINB distribution as the generating distribution $p_\theta(X|Z)$. Therefore, $\mathbb{E}_{Z\sim q_\phi(Z|X)}\left[\log p_\theta(X|Z)\right]$ in equation (S4) can be approximated as follows:

$$\mathbb{E}_{z\sim q_\phi(Z|X)}\left[\log p_\theta(x|z)\right] \approx \frac{1}{n}\sum_{i=1}^{m}\sum_{j=1}^{n}\log \mathrm{ZINB}(x_{ij}, \mu_{ij}, \theta_{ij}, \pi_{ij}). \tag{S5}$$

Then, we assume that $q_\phi(Z|X)$ follows a Gaussian distribution. For the second term in equation (S4), we can calculate it by Monte Carlo estimation:

$$\begin{aligned}
&-\mathrm{D_{KL}}(q_\phi(Z|X)\|p(Z)) \\
&= -\mathbb{E}_{Z\sim q_\phi(Z|X)}\left[\log q_\phi(Z|X)\right] - \mathbb{E}_{Z\sim q_\phi(Z|X)}\left[\log p(Z|X))\right] \\
&= \int q_\phi(Z|X)\log p(Z)\,\mathrm{d}Z - \int q_\phi(Z|X)\log q_\phi(Z|X)\,\mathrm{d}Z \\
&= \int N(Z;\mu_x,\sigma_x^2)\log N(Z;0,I)\,\mathrm{d}Z - \int N(Z;\mu_x,\sigma_x^2)\log N(Z;\mu_x,\sigma_x^2)\,\mathrm{d}Z \\
&= -\frac{d}{2}\log(2\pi) - \frac{1}{2n}\sum_{i=1}^{d}\sum_{j=1}^{n}((\mu_x)_{ij}^2 + (\sigma_x)_{ij}^2) - \left[-\frac{d}{2}\log(2\pi) - \frac{1}{2n}\sum_{i=1}^{d}\sum_{j=1}^{n}(1+\log(\sigma_x)_{ij}^2)\right] \\
&= \frac{1}{2n}\sum_{i=1}^{d}\sum_{j=1}^{n}(1+\log(\sigma_x)_{ij}^2 - (\mu_x)_{ij}^2 - (\sigma_x)_{ij}^2).
\end{aligned} \tag{S6}$$

Summarizing the above results, the loss function of the β-VAE network can be obtained as:

$$L_{\text{VAE}} = -\frac{1}{n}\sum_{i=1}^{m}\sum_{j=1}^{n}\log \text{ZINB}(x_{ij}, \mu_{ij}, \theta_{ij}, \pi_{ij}) - \frac{\beta}{2n}\sum_{i=1}^{d}\sum_{j=1}^{n}(1 + \log(\sigma_x)_{ij}^2 - (\mu_x)_{ij}^2 - (\sigma_x)_{ij}^2), \quad \text{(S7)}$$

where x_{ij} is the i-th row and j-th column of the matrix X; $\mu_{ij}, \theta_{ij}, \pi_{ij}$ are the i-th row and j-th column of the matrices M, Θ, Π of the ZINB distribution, respectively; $(\sigma_x)_{ij}^2$ and $(\mu_x)_{ij}$ are the i-th row and j-th column of the matrices σ_x^2 and μ_x (of the hidden layer Gaussian distribution), respectively.

Cluster splitting method

In the cluster initialization step of scAce, we include the option of clustering enhancement, which allows the user to start with cluster labels produced by another clustering method and obtain enhanced clustering results by optimizing the VAE network and cluster centroids. Since the existing clustering results from the other method may contain incorrect clusters with cells from multiple cell types, in order to maximize the enhancement, it is necessary to first split the initial clusters into purer subclusters.

Similar to the adaptive criterion used in the cluster merging step (see Methods), we split a previous cluster if its intra-cluster distance is greater than $\bar{d}/2$, where $\bar{d}$ is the average inter-cluster distance [2, 3]. Specifically, the intra-cluster distances of all previous clusters are calculated, and the cluster with the largest distance is split into two clusters if its intra-cluster distance is greater than $\bar{d}/2$. Then, the average inter-cluster distance, $\bar{d}$, is updated. The above splitting process is repeated until the intra-cluster distances of all clusters are smaller than $\bar{d}/2$.

When splitting a cluster into two smaller clusters, we use the Kaufman algorithm to identify the new centroids of the two clusters [4]. Suppose we would like to split cluster i, the algorithm works as follows.

1. For each cell in this cluster, we calculate the sum of its distances to all other cells in cluster i. The cell with the smallest sum is selected as the first centroid, and its index and coordinates are denoted as q_{i1} and c_{i1}, respectively.

2. For cells q and r in cluster i ($q, r \in N_i$ and $q, r \neq q_{i1}$), we calculate

$$T_{qr} = \max\{D_q - d_{qr}, 0\}, \quad \text{(S8)}$$

   where D_q is the distance between cell q and the first centroid, and d_{qr} is the distance between cells q and r .

3. We calculate $\sum_r T_{qr}$ for cell q ($q \in N_i$ and $q \neq q_{i1}$). The cell with the largest sum is selected as the second centroid.

After obtaining the two new centroids, the remaining cells in the cluster are assigned to their closer centroids to generate the two new clusters.

References

[1] Irina Higgins, Loic Matthey, Arka Pal, Christopher Burgess, Xavier Glorot, Matthew Botvinick, Shakir Mohamed, and Alexander Lerchner. beta-vae: Learning basic visual concepts with a constrained variational framework. In *International conference on learning representations*, 2017.

[2] Jingsheng Lei, Teng Jiang, Kui Wu, Haizhou Du, Guokang Zhu, and Zhaoqing Wang. Robust k-means algorithm with automatically splitting and merging clusters and its applications for surveillance data. *Multimedia Tools and Applications*, 75(19):12043–12059, 2016.

[3] Shisir Mia and Mohammad Motiur Rahman. An efficient image segmentation method based on linear discriminant analysis and k-means algorithm with automatically splitting and merging clusters. *International Journal of Imaging and Robotics*, 18(1):62–72, 2018.

[4] Leonard Kaufman and Peter J Rousseeuw. *Finding groups in data: an introduction to cluster analysis*. John Wiley & Sons, 2009.
